# Sensation seeking and risk adjustment: the role of reward sensitivity in dynamic risky decisions

**DOI:** 10.3389/fnbeh.2025.1492312

**Published:** 2025-02-07

**Authors:** Yin Qianlan, Chen Shou, Hou Tianya, Dong Wei, Taosheng Liu

**Affiliations:** Faculty of Psychology and Mental Health, Naval Medical University, Shanghai, China

**Keywords:** sensation seeking, risk adjustment, reward sensitivity, risky decisions, cognitive model, neural activity

## Abstract

**Objective:**

The primary objective of our research is to delve into the relationships between sensation seeking (SS), reward sensitivity (RS), and risk adjustment (RA) within the context of dynamic risk-taking behaviors. By integrating the reinforcement learning model and neural measures obtained from dynamic risk-taking tasks, we aim to explore how these personality traits influence individual decision-making processes and engagement in risk-related activities. We aim to dissect the neural and cognitive mechanisms underlying this interplay, thereby shedding light on the stable brain-based characteristics contributing to the observed variability in risk-taking and decision-making behaviors. Understanding these links could significantly enhance our ability to predict individual differences in risk preferences and develop targeted interventions for managing risky behaviors across different contexts.

**Method:**

We developed a task to measure RA through a structured yet uncertain environment modeled after the Balloon Analog Risk Task. We enlisted 80 young adults to perform this task, and of these, 40 were subjected to electroencephalography (EEG) to assess neural correlates of RS. Subsequently, we analyzed event-related potentials and spectral perturbations to discern neural distinctions related to RS. We compared these distinctions concerning RA among participants exhibiting different levels of SS.

**Results:**

Individuals exhibiting higher levels of SS (HSS) in the study displayed a tendency to disregard past risks, potentially resulting in diminished behavioral adaptability. EEG results indicated that individuals with HSS exhibited reduced neural responses to feedback compared to those with low SS, potentially affecting their feedback processing and decision-making. Moreover, the comparison of effects underscores the significant impact of RS and SS on shaping RA during dynamic decision-making scenarios.

**Conclusion:**

This study has advanced the understanding of how SS and RS influence RA, revealing that RS prompts RA, while individuals with HSS often exhibit blunted RS, leading to worse RA. Future research should focus on the specific aspects of HSS and their implications for decision-making across different risk contexts. Employing advanced neuroimaging and cognitive modeling techniques will be pivotal in unraveling the neural mechanisms driving these individual differences in risky behavior.

## 1 Introduction

Sensation seeking (SS) denotes individual tendency to seek diverse, new, intricate, and intense sensations and experiences, along with a willingness to take risks across various life domains for the sake of such experiences (Zuckerman, 2013). This characteristic is linked to engaging in real-world risky behaviors and experimental risky decisions. Some researchers have suggested that SS plays a direct role in risky decision-making by being associated with sensitivity toward reward during dynamic decision-making tasks involving uncertain rewards (Bornovalova et al., 2009; Aven and Renn, 2013). In scenarios involving risky decisions with uncertain rewards, reward sensitivity (RS) is connected to the assessment of particular rewards and may show variations in expectancy across different risk levels (Neuser et al., 2020). Research has demonstrated that SS can be both adaptive and maladaptive, depending on how it manifests and interacts with other personality factors. High levels of SS have been associated with various risk behaviors, including substance use and problematic internet use (Chase and Ghane, 2023). Moreover, RS is significant in certain psychiatric disorders, such as bipolar disorder and substance use disorders, which exhibit elevated levels of RS (Whitton et al., 2015; Kim-Spoon et al., 2016). Therefore, both SS and RS play a crucial role in the development of behavioral disorders.

Psychologists differentiate between SS and RS in the context of risky decision-making. SS is considered a stable personality trait, reflecting individual differences in the propensity to seek novel and intense experiences. RS, on the other hand, is viewed as a more state-like characteristic, varying within individuals based on their current motivational and emotional context (Fleeson and Jayawickreme, 2015). Research findings demonstrate that both aspects of personality contribute to molding individual disparities in decision-making processes. Individuals with high SS (HSS) tend to appraise risks lower than those with low SS (LSS). This difference is attributed to HSS individuals temporarily attenuating their attention to or sensitivity toward potential negative outcomes, while LSS individuals maintain a greater focus on potential losses (Horvath and Zuckerman, 1993; Lauriola et al., 2014). RS is thought to influence risk-taking by modulating the valuation of potential rewards or the impact of feedback during learning (Smillie et al., 2006). Hence, it could be understanded that SS may provide a stable framework for predicting risk-taking tendencies, while RS allows for a more nuanced understanding of how individuals may adjust their behaviors based on immediate circumstances and feedback. Importantly, they may be interconnected, such that RS could be a mechanism by which SS influences risk-taking behaviors (Bianca et al., 2023). However, limited empirical evidence exists for these interconnections.

Reward sensitivity is commonly evaluated in dynamic decision-making situations involving uncertain rewards and punishments, where individuals’ choices impact their sequential risk-taking behaviors. A key aspect of RS is reward responsivity, which refers to an individual’s responsiveness to rewards (Ross, 1975). Lower variation in reward responsivity corresponds to decreased RS (DiMenichi and Tricomi, 2016). Moreover, reward responsivity may influence individuals’ adjustment of their risk-taking behavior in response to feedback, as individuals are more likely to repeat rewarded actions and avoid unrewarded ones (Harmon et al., 2021; Lee et al., 2021; Markanday and Galarraga, 2021). The process by which individuals modify their risk-taking behavior based on feedback is also referred to risk adjustment (RA) (Iezzoni, 1997; Ahn et al., 2003; Juhnke et al., 2016; Kelley et al., 2019). RA is a key element in dynamic decision-making, illustrating how individuals flexibly adjust their risk preferences in response to past choice outcomes. Despite the recognized link between RA and RS, there is a scarcity of empirical research on RA in dynamic risky decision-making scenarios and its association with RS and SS.

Dynamic decision-making tasks provide a critical framework for understanding how individuals progressively develop behavioral strategies by systematically exploring the intricate relationships between SS, RS, and RA. By employing reinforcement learning (RL) models, particularly in contexts involving probabilistic monetary outcomes, researchers can effectively illuminate the complex mechanisms underlying reward processing and adaptive decision-making strategies (Botvinick and Braver, 2015; O’Doherty et al., 2017). RL methodologies formalize the acquisition of action values based on past experiences and elucidate the role of different valuation systems in decision control and the mechanism of reward anticipation (Sutton and Barto, 2018). They provide insight into how rewards or losses influence subsequent choice behavior and allow for an investigation into how RS shapes reward-driven behavior, particularly RA (De Wit et al., 2012; Patzelt et al., 2018). According to RL theory, learning is shaped by prediction errors (PEs) – essentially a type of unexpected result in comparison to the anticipated value, and determining this disparity (Sutton and Barto, 2018). These PEs update value expectations, shaping subsequent actions. Individual differences in RS likely moderate the impact of these PEs, effectively influencing the learning rate (Luman et al., 2012). Hence, a computational model incorporating RL principles could offer valuable insights into RA during dynamic risky decision-making. Specifically, such a model could elucidate the interplay between RS, as a state-like characteristic, and SS, a trait-like personality factor, in shaping risk-taking behavior.

Additionally, analyzing stable brain-based characteristics of a neural trait approach can partially account for this diversity in behavior (Nash and Knoch, 2016). Recently, investigations have suggested potential links between the rate of evidence learned from feedback and variations in the relative brain activity of RS via electroencephalography (EEG) (Frank et al., 2015; Fukunaga et al., 2018). Feedback-related negativity (FRN), an event-related potential component arising from the variance in electrical potentials between losses and gains, is sensitive to the valence of outcomes (Frank et al., 2015; Kim-Spoon et al., 2016; Nash and Knoch, 2016). It acts as a neural representation of reward PE and is responsive to discrepancies in reward probability and adjustments in posterior magnitude (Cohen and Ranganath, 2007; Walsh and Anderson, 2011). Furthermore, analyzing EEG data through time-frequency analysis to investigate reward processing has unveiled a wealth of insights. This method aids in distinguishing the distinct impacts of overlapping event-related potentials (ERPs) by segmenting the EEG signal into spectral power. Studies indicate that delta band activity (1–4 Hz) is especially responsive to rewards and positive reward PEs, whereas theta band activity (4–8 Hz) is primarily associated with negative outcomes and unsigned PEs (Sambrook and Goslin, 2016; Brown and Cavanagh, 2020). For these reasons, tracking neural activity during dynamic risky decision-making could contribute to illuminating state-based expression tendencies like SS and RS personality traits.

This article addresses the intricate connections among SS, RS, and RA through a RL framework and EEG signals from a dynamic uncertain decision task. We hypothesized that individuals with HSS would show a greater tendency to modify their choices due to deficiencies in intentional decision-making processes or generally exhibiting reduced preference for rewards. These anticipated findings might indicate that SS could have moderating influences on choice variability and reward, which relate to RA and RS.

## 2 Materials and methods

### 2.1 Participants

All participants in this research were students enrolled in a medical university. They were all right-handed individuals with normal or corrected-to-normal vision, devoid of any history of neurological or psychiatric disorders, head trauma, or recent alcohol or tobacco consumption within 2 weeks before the study. The experimental protocols were endorsed by the Research Ethics Committee of Second Military University, Shanghai, China. Each participant provided written informed consent, acknowledging the objectives and methodologies of the study. There were no anticipated risks or discomfort, and participants were compensated upon task completion.

In study 1, 44 participants completed the task in our behavior lab using computers without EEG equipment. In study 2, 44 college students who did not participate in study 1 performed the task in the behavior lab while wearing EEG caps. However, one participant withdrew from study 1, and seven participants from study 2 were excluded from subsequent analyses due to technical issues or movement artifacts during data collection.

### 2.2 Procedure

Initially, all participants were invited to our laboratory and instructed to complete paper questionnaires, which included assessments of personality traits and demographic details, after providing written informed consent. Following a briefing on the safety protocols associated with EEG devices, participants engaged in computer-based tasks using the Windows 7 operating system and E-prime 2.0 software within a noise-reduced psychology laboratory environment, positioned approximately 60 cm away from a 20-inch LCD screen. Notably, participants in study 2 undertook the task while wearing a 64-channel EEG cap (Biosemi Product) with conductive gel. Both sets of participants completed the tasks within the same laboratory setting.

The task duration in study 1 averaged 15 min without fixed intervals between interfaces. In contrast, in study 2, the task lasted no less than 45 min due to the EEG setup prolonging interface delays and incorporating intermediate breaks. Before commencing the experiment, all participants underwent 15 practice trials, during which they carefully reviewed the task instructions and familiarized themselves with the risk characteristics. Participants wearing EEG caps were instructed to maintain stable head positions, focus on the screen, and minimize muscular movements. However, occasional glances at the keyboard for responses were inevitable, prompting a brief task delay to refocus attention on the screen. Following this delay, feedback on task performance was provided. Upon task completion, participants received compensation proportional to their risk decision-making performance, with an average payment of approximately 100 yuan for all participants and a standard compensation of 100 yuan for participants in study 2 to meet the experimental requirements.

### 2.3 Sensation Seeking Scale

The Sensation Seeking Scale Version 5 (SSS-V) is a psychometric tool that assesses individual variations in the desire for novel experiences and sensations (Zuckerman, 2007). This scale can evaluate both enduring personality traits and temporary states of SS, making it applicable to individuals across various age groups, including adults and adolescents, and suitable for diverse settings such as clinical, research, and educational environments (Zuckerman and Aluja, 2015). Widely recognized for its reliability and validity in measuring SS, the SSS-V has been extensively utilized in research to investigate the correlation between SS tendencies and a range of behaviors and outcomes (Zuckerman, 2015).

In this study, the Chinese version of the Sensation Seeking Scale Form V (SSS-V) was utilized and completed by all participants before engaging in the behavioral assessments. This scale comprises four sub-scales, each comprising 10 items: thrill and adventure seeking, boredom susceptibility, experience-seeking, and disinhibition (Wang et al., 2013). The total score is derived by summing the responses to all 40 items, providing an overall SS score reflecting the combined contributions. The scale’s internal consistency, as indicated by Cronbach’s alpha, was calculated to be 0.805, demonstrating good reliability. Detailed results of the interrelationships between sub-scales and the outcomes of factor analysis can be found in the Supplementary material.

### 2.4 Dynamic decision-making task (Balloon Inflation Test)

**FIGURE 1:**
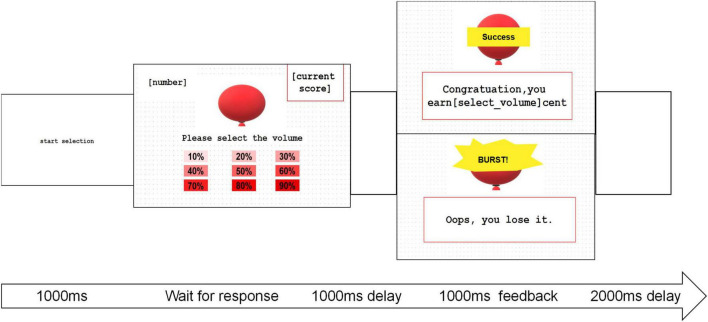
The diagram depicting BIT with consistent intervals between interfaces. In the balloon inflation task, participants’ focus was directed by the “start choice” phase lasting 1,000 ms, allowing them to choose the degree of inflation. Subsequently, an empty screen was displayed for 1,000 ms, followed by feedback lasting 1,000 ms. A 2,000 ms interval with a blank screen followed before initiating the next trial.

The Balloon Inflation Test (BIT) was derived from the Balloon Analog Risk Task (BART) (Lejuez et al., 2002). The BIT entailed a calculated risk assessment to quantify RA and incorporated more sophisticated measures for observing behaviors. The task was structured with fixed intervals between interfaces, as illustrated in Figure 1. Participants were briefed that they could earn money by inflating balloons, with the earnings directly linked to the chosen inflation percentage. Following a practice session, participants made trial-by-trial adjustments to optimize their earnings. Each choice corresponded to a specific likelihood of the balloon bursting, and rewards were determined based on the degree of inflation chosen. While the quality of the balloons was randomly generated and undisclosed to the participants, statistically, the optimal choice at the group level was 50%. Behavioral data from each trial, encompassing responses, reaction times, feedback, losses, and earnings, were meticulously recorded using E-prime 2.0 Professional.^1^ After completing the 60 trials, participants were queried about their preferred inflation range by selecting options such as “1 – among 10%–30%,” “2 – among 30%–50%,” “3 – among 50%–70%,” and “4 – among 70%–90%.”

### 2.5 Electroencephalography recording

During the BIT, EEG recordings were conducted using the Biosemi ActiveTwo amplifier system (Biosemi, Amsterdam, Netherlands) with a bandpass of 0.1–100 Hz and a sampling rate of 512 Hz. This system utilizes two active electrodes, the Common Mode Sense (CMS) and Driven Right Leg (DRL), in place of traditional “ground” electrodes. The CMS is a recording reference, while the DRL serves as ground. Participants wore a flexible electrode cap with 64 Ag/AgCL electrodes arranged according to the International 10–20 system. EEG data were initially recorded using nasorostral as an online reference and then re-referenced algebraically to the average of all 64 channels for each participant. Electrode impedance was maintained below 3 kΩ. Horizontal and vertical electrooculograms were captured to detect blinks and eye movements. Subsequently, all data were processed offline using MATLAB R2020a (Math Works, Natick, MA) and the EEGLAB Toolbox 12.0.1 (Delorme and Makeig, 2004). A 30-Hz low-pass filter and a 0.1-Hz high-pass filter were applied for data filtering. Each EEG epoch commenced 1,000 ms before the feedback onset and concluded 1,000 ms after onset. Prior to averaging, independent Components Analysis was employed to correct for eye-blink and movement artifacts. Any trials contaminated by eye movements, blinks, or muscle potentials exceeding ±100 μv at any electrode were excluded from the analysis.

## 3 Statistical analysis

### 3.1 Behavioral data processing

In the context of the BIT, it is crucial to assess the average inflation level and its variation to comprehend participants’ risk-taking behavior. Analyzing the average inflation level provides insight into general risk inclination while studying inflation variability, which can yield valuable information on decision-making processes and response dynamics during the task. Additionally, a detailed examination was conducted on a trial-by-trial basis to improve understanding of decision precision, learning effects, and behavioral dynamics observed in the experimental data. This approach involved treating the number of trials as a fixed-effect factor and examining its influence on inflation choices based on sensation-seeking levels. Linear mixed-effects modeling using R’s lme4 package was conducted to conduct this trial-by-trial analysis. Furthermore, these effects were visualized using the ggplot2 package for comprehensive interpretation.

### 3.2 Computational modeling

To characterize the learning behavior of participants in BIT and reveal underlying trial-by-trial aspects of decision-making variables, we developed two sets of computational models. We applied them to the behavioral data of participants. These models were based on the simple RL model following the Rescorla-Wagner (RW) learning rule, as well as the RL model incorporating a Kalman (KL) filter (Rescorla, 1972). We assessed the suitability of these models and progressively integrated the most effective ones by comparing their performance. Within the RL model framework, each decision was represented by a 9-option value spanning from 10% (*V*_*t*(1)_) to 90% (*V*_*t*(9)_):


(1) $${\tilde{V}}_{t}=[V_{t}\left(1\right),V_{t}\left(2\right),V_{t}\left(3\right),V_{t}\left(4\right),V_{t}\left(5\right),V_{t}\left(6\right),V_{t}\left(7\right),$$

$$V_{t}\left(8\right),V_{t}\left(9\right)]$$

In each trial *t*, *V*_*t*_ (representing the option’s value) was depicted as a two-element vector with a value of zero (indicating not chosen) or one (indicating chosen). Given the nature of the task, where each option was associated with increasing rewards, the likelihood of each option remaining at its current level or advancing to a higher degree was considered. For example, if a participant selected 50%, they were expected to choose 40% in the next trial, indicating a gradual progression in their decision-making. This aligns with the notion that participants were inclined to advance to higher degrees cautiously, as evidenced by their reluctance to jump directly to 70% without first embracing the risk of 60%. This decision-making process was reflected in the probability of choosing option i (indexed from 1 to 9), as outlined below.


(2) $$P_{t}\,\left(i\right)=\frac{e^{V_{t}\,\left(i\right)}}{e^{V_{t}\,\left(i\right)}+e^{V_{t}\,\left(i+1\right)}}=\frac{1}{1+e^{-\left(V_{t}\,\left(i\right)-V_{t}\,\left(i+1\right)\right)}}$$

The values were subsequently transformed into action probabilities through the utilization of a SoftMax function:


(3) $$P_{t}\,\left(i\right)=\Phi \,\left(V_{t}\,\left(i\right)\right)$$

Where Φ was the inverse logit linking function:


(4) $$\Phi \,\left(x\right)=\frac{{\text{e}}^{\text{x}}}{1+{\text{e}}^{\text{x}}}=\frac{1}{1+{\text{e}}^{-\text{x}}}$$

It is important to note that we incorporate the widely employed inverse SoftMax temperature parameter τ in the model specifications of action probability. This parameter regulates the degree of randomness in decision-making, varying from τ = 0 for entirely random responses to τ = ∞ for selecting the highest value option with certainty. In the basic model, an RW model was employed to represent decision-making, where only the selected value was adjusted based on the PE. In contrast, the unselected value remained unchanged from the previous trial.


(5) $$P\,E=R_{t}\,\left(i\right)-V_{t}\,\left(i\right)$$

(6) $$V_{t+1}\,\left(i\right)=V_{t}\,\left(i\right)+\alpha \,P\,E$$

Here, *R*_*t*_ represented the reward received in trial *t*, and the learning rate (α_t_) denoted by RS (0 < α_t_ 1) determined the influence of the PE on updating the value. Upon inputting values of (1), (2), (3), (4), and (5), we obtained the categorical distribution of choices as:


(7) $$C\,h\,o\,i\,c\,e\,C\,a\,t\,e\,g\,o\,r\,i\,c\,a\,l\,\left(\Phi \,\left(\tau \times V_{t}\right)\right)$$

The model incorporating the KL filter, known as the Pearce-Hall model, is additionally computed with the standard error (Gheza et al., 2018). This error signifies the dynamic learning rate that governs the impact of the PE and employs a form of the delta rule to adjust the estimated value according to the reward PE. KL filter models stand out by monitoring the (posterior) variance of the estimated value for each choice, reflecting estimation uncertainty, and leveraging this information to modify the learning rate adaptively. The lazy KL filter introduces a bias to the learning rate, facilitating a slower learning process. This model introduces additional parameters: the initial learning rate and the asymptotic learning rate, which collectively characterize the progression of the effective learning rate over time. The term “KL gain” represented by *k*_*t*_ functions as a learning rate. Consequently, Equation 6 was revised as:


(8) $${\text{V}}_{\text{t}+1}\,\left(i\right)={\text{V}}_{\text{t}}\,\left(i\right)+k_{t}\,\alpha_{\text{t}}\,\text{PE}$$

The parameter η ? (0, 1) dictates the bias in the updates of the KL gain, potentially leading to a slower learning rate (hence the term “lazy”). In the standard KL filter, this parameter is set at η = 1, whereas in the lazy versions, it is a variable parameter, allowing for less precise updates. The term *k*_*t*_ is influenced by *S*_*t*_, representing the variance of the posterior distribution of the average reward, incorporating the innovation variance and the reward variance of the option. When *t* = 1, it signifies the initial variances as priors. These concepts are outlined as follows:


(9) $$\alpha_{\text{t}+1}=\eta \,\left|\mathrm{PE}\right|+\left(1-\eta \right)\,\alpha_{\text{t}}$$

(10) $$k_{t}=\frac{S_{t}+\sigma_{\zeta }^{2}}{S_{t}+\sigma_{\zeta }^{2}+\sigma_{\varepsilon ,t}^{2}}$$

In our models, logistic regressions are commonly utilized for vectors, and we employed the logistic model due to the U-shaped pattern observed in the expected values in our study. Specifically, the choice of 30% was rewarded equivalently to 70% based on probability. Consequently, we postulated a non-continuous computational process of choice utilities when individuals were making choices, aligning with the fundamental assumption of the logistic regression model. In this analysis, we initialized the values of *V*_*t*_(*i*) at 0, while the subsequent choices, V1–V9 (representing the utility of the 9 options calculated as the product of risk probability and reward), were 9, 16, 21, 24, 25, 24, 21, 16, and 9, respectively. The standard deviations derived from reward probability were 3.12, 4.22, 4.83, 5.16, 5.27, 5.16, 4.83, 4.22, and 3.12, respectively.

We evaluated the superior model against alternative computational hypotheses within the hierarchical Bayesian framework (Table 1). To further validate our leading model, we employed two rigorous approaches. Firstly, we conducted a parameter recovery analysis to ensure the accurate and specific identification of all parameters (refer to Supplementary material). We conducted posterior predictive checks by leveraging model comparison to assess relative model performance. These analyses were executed in the R environment utilizing a combination of Rstan for Bayesian inference, hBayesDM for hierarchical Bayesian modeling, and loo for model comparison. The distinction in parameters was assessed using the Wilcoxon Rank Sum Test.

**TABLE 1:** Computational models, model parameters, and model comparison.

Model	Parameter	Prior	Hyperpriors		LOOIC
RW-RL	Learning rate (α)	Φ (μ_α_ + ζ_α_ ν_α_)	μ_α_ ∼Normal	ζ∼Half-Normal	17,413.6 ± 379.0
	Inverse temperature (τ)	exp (μ_τ_ + ζ_τ_ ν_τ_)	(−1, 1) μ_τ_ ∼Normal (0, 1)	(0, 1) ν∼Normal (0, 1)	
KL-RL	The innovation variance ($\sigma_{\xi }^{2}$)	exp (μ$\sigma_{\xi }^{2}$ + ζ $\sigma_{\xi }^{2}\,\nu \,\sigma_{\xi }^{2}$)	μ∼Normal (0, 1)		17,383.6 ± 380.7
	Initial variance ($\sigma_{\varepsilon }^{2}$)	exp (μ$\sigma_{\varepsilon }^{2}$ + ζ$\sigma_{\varepsilon }^{2}\,\nu \,\sigma_{\varepsilon }^{2}$)	μ∼Normal (2, 1)		
	The bias of KL gain (η)	Φ (μ_η_ + ζ_η_ ν_η_)	μ_η_ ∼Normal (1, 1)		
	Inverse temperature (τ)	exp (μ_τ_ + ζ_τ_ ν_τ_)	μ_τ_ ∼Normal (0, 1)		

All models employ non-centered reparameterization as specified in the prior column, frequently transformed to restrict parameters to non-negative values (exp) or within a specific range (Probit function, Φ). LOOIC refers to the leave-one-out information criterion concerning the winning model (a lower LOOIC value signifies enhanced out-of-sample predictive accuracy). The group level is denoted by μ, with v representing the individual level, and ζ indicating the standard error of the samples.

### 3.3 Event-related potentials and spectral perturbations analysis

For each EEG epoch, we established the baseline for ERPs measurements by averaging the voltage recorded during the 200 ms pre-feedback interval and 1,000 ms. Subsequently, FRN was evaluated by calculating the mean wave amplitudes within a time window of 250–320 ms following positive or negative feedback onset. In line with previous studies, the FRN was initially evaluated across three electrodes: Fz, FCz, and Cz, prompting a focused and detailed analysis on these specific electrodes (Euser et al., 2011; Takács et al., 2015). Therefore, our detailed analyses were centered on the Fz, FCz, and Cz electrodes. Additionally, we conducted a time-frequency analysis on single-trial ERP data synchronized with feedback. This analysis involved averaging and baseline correction to ascertain event-related spectral perturbations (ERSPs). The algorithm used for time-frequency analysis is based on the Morlet wavelet transform principle involving convolution between wavelets with peak frequencies and the temporal signal under investigation to obtain a representation of power at different frequencies within the temporal domain signal. The dataset examines a frequency range from 0.1 to 30 Hz in the frequency domain divided into 50 frequency points. The temporal width spans 2 s, encompassing the entire epoch, with a margin of ±1 s around the presentation of the result feedback. The wavelet cycles range from 3 to 14, and trial-specific time-frequency results undergo averaging and normalization processes. To reveal the effect of SS on RS related neural substrates, we compared the difference in these related substrates using the Wilcoxon Rank Sum Test

### 3.4 Moderation effect statistic

The moderation effect statistic was computed to examine the interaction between SS and RS on RA measured in the dynamic risky decision. We used multiple regression analysis to test the interaction effect, with SS and RS as predictors and the choice degree varied across bins as the outcome variable with choice order as the intercept. The interaction between SS and RS can be defined mathematically as:


(11) $$Y_{i\,j}=\beta_{0}+\beta_{1}\,S\,S_{i}+\beta_{2}\,R\,S_{j}+\beta_{3}\,\left(S\,S_{i}\,R\,S_{j}\right)+\gamma_{k}+{?}_{i\,j}$$

In this context, *Y* is the choice degree varied across trials, and β_3_ signifies the interaction effect that demonstrates the varying impact of RS on the outcome, in response to the dynamic risk associated with RA, based on the level of SS. By including random effects, mixed models can handle correlations within the data and provide more accurate estimates of fixed effects. The analyses were performed in R with the “lmer” function. Additionally, we verified the centering of all variables in the model using the R functions (scale).

## 4 Results

### 4.1 Demographic characteristics

A summary outlining the demographic features of all participants is displayed in Table 2. The collective average SS scores stood at 99.63, with a standard deviation of 13.23. As there were no notable distinctions in the behavioral outcomes (*P*s > 0.055), we simultaneously evaluated participants’ behavioral performance across both studies.

**TABLE 2:** Sample demographic characteristics, SS scores (SSS), and the averages of behavioral variables.

Characteristic	Total	Study 1	Study 2	*P*-value
	***N* = 80**	***N* = 43**	***N* = 37**	
Gender				0.096
Male	27 (34%)	11 (26%)	16 (43%)	
Female	53 (66%)	32 (74%)	21 (57%)	
Age	21.39 ± 3.13	21.16 ± 2.92	21.65 ± 3.38	0.442
SSS	99.63 ± 13.23	95.58 ± 11.99	104.32 ± 13.20	0.003
Mean choice (%)	46.26 ± 8.27	45.70 ± 8.56	46.91 ± 7.98	0.689
Mean RT	1,215.09 ± 493.32	1,261.78 ± 693.11	768.93 ± 636.07	0.828
Choice standard deviation	17.16 ± 5.01	18.15 ± 4.97	16.00 ± 4.86	0.055
Choice counts of 50%	15.96 ± 11.88	15.74 ± 11.31	16.22 ± 12.66	0.973
Attitude				0.040
10%∼30%	26 (33%)	14 (33%)	12 (32%)	
30%∼50%	46 (58%)	28 (65%)	18 (49%)	
50%∼70%	8 (10%)	1 (2.3%)	7 (19%)	

Cell were presented as number (%) or mean ± SD. Behavioral variables include the mean of chosen degree (abbreviated as Meanchoice), mean of reaction time (Mean RT), choice variability (choice standard deviation), times of choosing 50% (choice counts of 50%).

### 4.2 Risk adjustment behavior among individuals with varying level of SS

**FIGURE 2:**
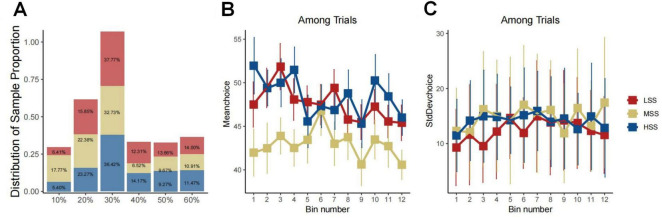
Group-behavioral characteristics of LSS, MSS, and HSS groups. Panel **(A)** presents a bar graph illustrating the correlation between the distribution of sample proportions and choices. The bars are color-coded to represent distinct groups, with each bar labeled by its corresponding percentage, signifying the proportion of each choice within the group. Panels **(B,C)** depict three-line graphs, evidently linked to a time series of experiments. The *x*-axis denotes the experimental stage with increments of five trials, grouped into bins. Meanwhile, the *y*-axis portrays the mean and standard deviation of choices over five trials. In both graphs, different colored lines signify data from diverse groups. Error bars denote the variability or uncertainty of the measured points within the bin.

At the initiation of our analysis, we investigated the associations between the Sensation Seeking Scale (SSS) scores and the behavioral indexes derived from the BIT. The findings revealed predominantly insignificant correlations between SSS and its subscale scores with BIT indexes, including reaction time, variability, and maximum scores (refer to the Supplementary material). To further explore the relationship between different levels of SS and their impact on BIT performance, we conducted a three-group analysis, categorizing participants into high (HSS), medium (MSS), and low (LSS) sensation seeking groups based on the 33rd and 66th percentiles of the SSS scores considering a potential nonlinear relationship between SSS and BIT results. This allowed us to more meticulously explore the relationship between different levels of SSS and behaviors. During the grouping process, we ensured a balanced amount of data in each group to guarantee the accuracy and reliability of the results. This resulted in 27 participants being placed into the LSS group, 30 into the MSS group, and the remaining 23 into the HSS group. To further explore behavioral differences between these groups, we analyzed their choice details as depicted in Figure 2. The figure illustrates similar choice distributions for LSS and HSS groups, while MSS preferred lower risk over other groups (χ^2^ = 27.94, df = 2, *P* < 0.0010, see Figure 2A). The regression results also indicated that trial orders affected choice preferences (β = −0.339, SD = 0.127, *P* = 0.007, see Figure 2B) and being in the MSS (β = −6.383, SD = 2.444, *P* = 0.010, see Figure 2B). However, there was no significant difference in the choice of standard deviation among these groups (see Figure 2C).

### 4.3 Estimation and comparison of parameter values for RS

**FIGURE 3:**
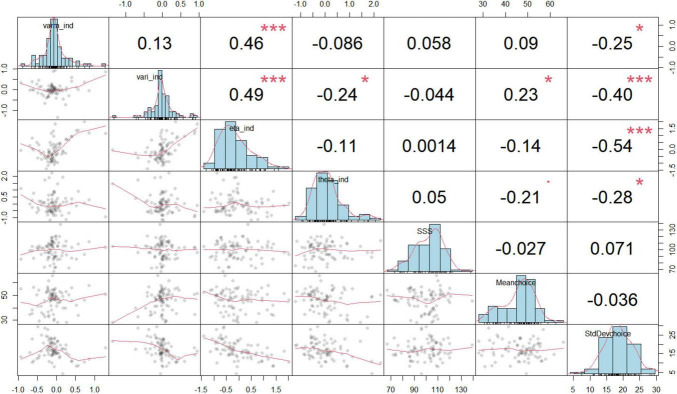
Relationships between model parameters and behaviors. Each of the small plots in the lower triangle of the matrix is a scatterplot of two variables. The plots on the diagonal are histograms showing the distributions of individual variables. Above the diagonal, there are correlation coefficients. The histogram visually displays the occurrence frequency of values for each variable at the individual level, encompassing the bias of KL gain (eta_ind), inverse temperature (theta_ind), the innovation variance (varm_ind), and initial variance (vari_ind), the mean of the selected degree (Meanchoice), the mean reaction time (MeanRT), the variability in choices (standard deviation of choices, abbreviated as StdDevchoice), and the frequency of choosing 50% (choice counts of 50%). The symbols in this figure represent statistical significance levels: * indicates *p* < 0.05; ** indicates *p* < 0.01, *** indicates *p* < 0.001.

We collectively inputted the data from the three groups into the Hierarchical Bayesian models to uncover the distinction at a more detailed level. Utilizing the Markov Chain Monte Carlo (MCMC) method, we performed a comprehensive simulation of data, primarily aimed at estimating the range of parameters as posterior distributions for each parameter at the individual level (Gallagher et al., 2009). The specifics of each hierarchical Bayesian model were outlined in Supplementary material. Based on the leave-one-out information criterion (LOOIC) presented in Table 1, the KL-RL model exhibited slightly superior predictive performance compared to the RW-RL model. Consequently, we concluded that the parameters of the KL-RL model were more effective in representing RS during the learning process. Figure 3 displayed the connections among model parameters, demonstrating a negative correlation between model parameters and choice standard deviation.

**FIGURE 4:**
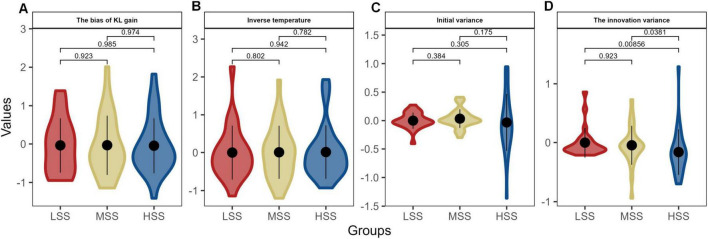
The posterior distributions for individual parameters of KL_RL model. Panels **(A–D)** depict the model parameters distributions in the three groups, respectively. The violin plots combine elements of a box plot with a kernel density estimation. The thick black line inside each colored shape shows the interquartile range (IQR), and the white point represents the median. The width of the colored shape at different points on the *y*-axis shows the probability density of the parameter values: the wider the section, the higher the probability of the parameter taking a value within that range. The different colors per group allow for a quick visual comparison across groups.

Hence, we separately entered the behavior data of three groups into the three hierarchic models, and the posterior distributions for the bias of KL the bias of KL gain (η, Figure 4A), inverse temperature (τ, Figure 4B), the innovation variance ($\sigma_{\xi }^{2}$, Figure 4C), and initial variance ($\sigma_{\varepsilon }^{2}$, Figure 4D) were calculated at the individual level. As depicted in Figure 4, only one parameter showed significant differences among the groups with varying levels of SS. The HSS group exhibited the highest value, which was significantly different from the LSS and MSS groups (*P*s < 0.038); however, there was no significant difference between the MSS and LSS groups (*P* = 0.923). These findings suggest that individuals with HSS may have a stronger inclination toward unfamiliar risks and show persistence in their decision-making.

### 4.4 The correlation between SS and neural substrates of RS

**FIGURE 5:**
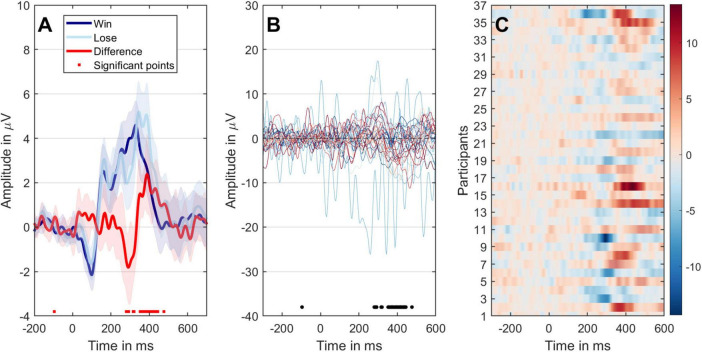
The different representations of the ERP data of two conditions. **(A)** Mean ERPs with 95% confidence intervals. The black dots on the *x*-axis indicate time points with significant paired *t*-test results (*P* < 0.05). The dark-blue line represents the win condition, the light-blue line represents the loss condition, and the red line signifies the comparison between loss and win conditions. **(B)** The temporal evolution of ERP variances between the two conditions for individual participants is displayed. **(C)** The progression of individual differences over time, with participants arranged along the *y*-axis. ERP amplitudes are color-coded to correspond with the outcomes depicted in panel **(B)**. Significant time points are highlighted with transparency. A bootstrap cluster sum method was employed to adjust for multiple comparisons.

In BIT, successful inflation results in a “Win” and corresponding scores, while bursting the balloon leads to a “Loss.” Therefore, measuring RS is based on the differences in event-related potentials recorded from Fz\FCz\Cz channels. Figure 5A illustrates significant variations between the two conditions at time points around 250–450 ms, evident in overall ERPs and individual participant trial differences. Moving forward to Figure 5B, it seems that participants’ trial differences reach their peak latency around 300 ms, further supported by statistical analysis shown in Figure 5C. A detailed analysis of ERP differences and their correlation with SS was presented in Supplementary material. This analysis demonstrates that the time window between approximately 250–450 ms exhibits significant differences in the grand-averaged ERPs between the two experimental conditions.

**FIGURE 6:**
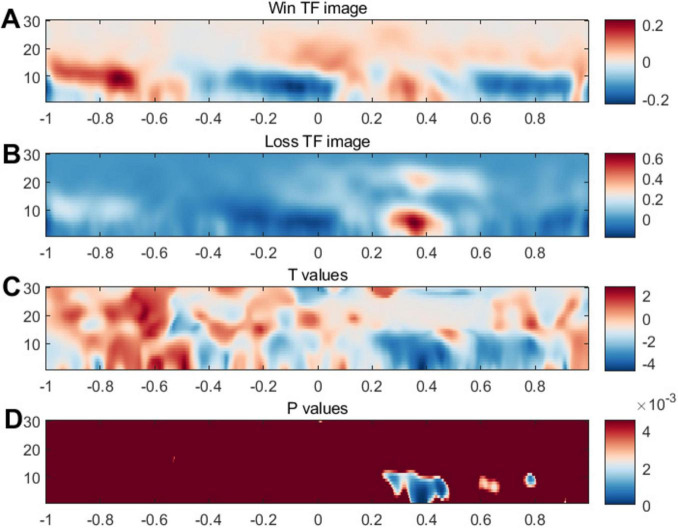
Event-related synchronization related to feedback. Panels **(A,B)** illustrates brain activity’s intensity or synchronization levels. The color scheme typically indicates intensity or power, with blue possibly representing lower synchronization or power and red indicating higher synchronization or power. Panel **(C)** may present a T-contrast of the (Win vs. Loss) conditions. Panel **(D)** displays *P* values, a measure of statistical significance; the blue areas indicate time points and frequencies where the data significantly differ from background or control conditions.

Moreover, we conducted ERSP analysis focusing on the signals between 250 and 450 ms. Figure 6 illustrates the ERS in the 1–30 Hz frequency range within the notable time-frequency window. Consistent with the ERP outcomes, we observed heightened power at FCz following the feedback, aligning with earlier findings related to FRN. In Figure 6D, statistical significance was evident within the 200–400 ms timeframe and across frequencies spanning 1–10, showing notable differences in the comparison at the low- and middle frequency bands. Additionally, topographic maps for the ERSP results, contrasted across different channels, were provided in the Supplementary material.

**FIGURE 7:**
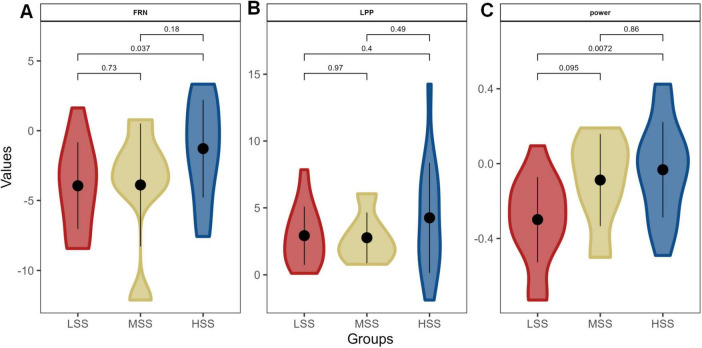
The difference of neural substrates for RS among groups with different levels of SS. Panels (**A–C**) depict the amplitude of the feedback-related negativity (FRN), the late positive potential (LPP) amplitude, and the power within the 1-10 Hz frequency band, respectively.

To demonstrate the impact of SS on RS concerning neural pathways, we compared individuals with varying levels of SS. This comparison revealed significant differences between high and low levels of SS in terms of both the FRN amplitude (*P* = 0.037, Figure 7A) and the power within the 1-10 Hz frequency band (*P* = 0.0072, Figure 7C). No significant difference showed in the LPP amplitude (Figure 7B). Individuals with HSS exhibited smaller amplitudes and weaker responses to negative feedback than those with LSS.

### 4.5 The moderating effect of SS on RS and RA

Three regression models were constructed to examine the moderate impact of SS (Table 3). These models used RA to represent the variation in choice degree as the outcome variable. The random effect was defined as choice order (categorized 10 times), and the common fixed effect was represented by levels of SS. Model1 included the individual-level model parameter ($\sigma_{\xi }^{2}$) and tits interaction with SS from study 1 and 2 samples. In model 2 and 3, FRN, band power, and their interaction with SS were the predictive variables with samples from study 2. When compared to the HSS group, it was observed that the interaction between SS and FRN significantly predicted variations in choices across bins, indicating that at higher levels of SS, the positive predictive effect of FRN on choice variation became stronger.

**TABLE 3:** Summaries of effects on RA.

Predictors	Estimates	CI	*P*	Estimates	CI	*P*	Estimates	CI	*P*
(Intercept)	48.44	46.99–49.90	<0.001	55.47	52.72–58.22	**<0.001**	50.63	47.70–53.56	**<0.001**
Group (LSS)	−1.12	−3.11 to 0.86	0.267	−4.19	−8.59 to 0.20	0.061	−0.73	−4.76 to 3.29	0.72
Group (MSS)	−4.65	−6.67 to −2.63	**<0.001**	−10.13	−13.24 to −7.02	**<0.001**	−6.17	−9.44 to −2.89	**<0.001**
varm ind	−0.82	−6.68 to 5.03	0.783						
Group (LSS) × varm ind	−4.8	−11.96 to 2.36	0.189						
Group (MSS) × varm ind	6.14	−0.63 to 12.91	0.075						
FRN				1.64	1.11–2.17	**<0.001**			
Group (LSS) × FRN				−1.16	−1.98 to −0.33	**0.006**			
Group (MSS) × FRN				−0.76	−1.46 to −0.06	**0.033**			
Power							5.41	−2.23 to 13.06	0.165
Group (LSS) × power							0.16	−13.91 to 14.23	0.982
Group (MSS) × power							1.65	−8.45 to 11.74	0.749

The dependent variable was the choice degree varied across bins. The random effect was defined as choice order (categorized 10 times), and the common fixed effect was represented by the level of SS using HSS as the reference. The significant *p*-values have been highlighted in bold.

## 5 Discussion

As we predicted, SS may have a moderating influence on choice variability, demonstrating RA through RS. This was confirmed by the RL model method and further supported by neural substrates showing the relationship between SS and RS. Regarding behavioral findings, individuals with HSS are likely to display a decreased tendency to alter choices and low levels of RS in response to reward feedback. Additionally, EEG results revealed a significant effect of SS on the neural substrates of RS. In particular, FRN related to RS predicted RA, and its effect was moderated by SS. These results highlight SS’s behavioral presentation and neural substrates and contribute to understanding the complex interplay between SS and RS in influencing RA.

Previous studies also found that SS and RS are closely connected in terms of the pursuit of reward and novelty (Zuckerman, 1994; Bornovalova et al., 2009; Xu et al., 2019). HSS individuals are more sensitive to rewards but less sensitive to punishment compared with LSS. The potential explanation centers around motivation in SS and the “hyperactive approach system” (Kruschwitz et al., 2012). HSS might be inversely linked to RS, as it may require a higher degree of risk to meet the seekers’ presumed excitement and arousal for reward (Zuckerman, 2007). In particular, we made an unexpected observation after dividing the comparison groups into three levels, which differs from traditional pairwise comparisons. Our observations indicated that the MSS group demonstrated a decreased tendency for risk compared to the HSS group. This indicates that the MSS group may lead to a lower preference for risk-taking. However, these distinctions are only apparent in the average inclination and do not persist in choice variability. These results indicate a difference in the relationship between SS and risk inclination compared to SS and RA. Advancedly, we used an RL model combined with a KL filter to investigate the potential impact of SS on RA. We also incorporated the influence of RS using relevant parameters. The KL filter’s ability to handle uncertainty and evolving situations makes it ideal for examining the effects of SS and RS on RA in decision-making processes involving learning from feedback (Zhang et al., 2021; Kim et al., 2022). Through model analysis, we found that individuals with HSS tend to disregard past risks and concentrate excessively on immediate stimuli and potential rewards, causing them to neglect previous encounters with risks. This behavioral pattern can impact their adaptability and decision-making flexibility in dynamic environments.

Reward sensitivity has evolved from being a trait related to the behavioral approach and inhibition system to being seen as a state personality that can be applied in human behavioral experiments (Kale et al., 2018; Fischer and Karl, 2020). However, most field studies used self-reported surveys, with only a few investigating the relationship between state and trait personality in experimental behavioral tasks, especially those related to risky decision-making. As a result, our research utilized an experimental task to control the outcome of a risky decision. Subsequent choices were influenced by outcomes that likely relate more to feedback or reward-based learning processes. This served as a practical measure for assessing how personalities affect decision outcomes. Similar to previous studies, we discovered that RS (the tendency to repeat previously rewarded actions while avoiding losses) played a pivotal role in shaping decisions during reward-based learning and decision-making situations (Cockburn and Holroyd, 2018). RS directly links to individual learning rates that scale the effect of PEs on updating values (Wu et al., 2017). These concepts extend to environments involving dynamic uncertain decisions, where RS involves comparing a choice’s expected value with its actual outcome – signaling subjective-value representation or “satisfaction” with a choice’s result. These results suggest that RS affects how risk is evaluated and adjusted afterward, which implies that people who are highly sensitive to rewards tend to take more risks when making decisions and adapt their risk preferences based on their experiences. Hence, this emphasizes the importance of RS in shaping adjustments made for risky decisions in dynamic environments.

Examining consistent brain-based traits in individuals using a neural trait approach can help explain some of the variations in behavior (Nash and Knoch, 2016). We aimed to investigate further the underlying neural mechanisms of SS and its influence on RS-related behaviors. We employed EEG to examine the brain activities associated with RS during a reward-based decision-making task to achieve this. Our findings indicated that individuals with HSS showed reduced responsiveness to different types of feedback compared to those with LSS. Consistent with our observations regarding differences in FRN, previous studies have demonstrated that the magnitude of FRN changes based on learning from PEs generated by RL models (Walsh and Anderson, 2011). FRNs have been associated with risky decision-making behavior (Polezzi et al., 2010; Takács et al., 2015; Zhong et al., 2020). The emergence of an active FRN signal during such decisions may reflect a conflict between expected and actual outcomes, leading to emotions related to regret or disappointment. This suggests that features defining FRNs make them valuable indicators for monitoring neural activity during dynamic risky decision-making, offering insights into overarching pattern analysis and potentially shedding light on state-based expression tendencies such as SS and RS personality traits. Our study provided supporting evidence for these aspects of the value of FRN.

Besides, time-frequency analysis revealed that individuals with HSS exhibited increased theta band power for reward in the prefrontal cortex during decision-making, suggesting their heightened cognitive processing and attention allocation to reward-related information. Consistent with prior findings, the links between theta (4∼8 Hz) signals associated with cognitive control and adjustment of thresholds (Cavanagh et al., 2010; Narayanan et al., 2013), as well as associations between trial-to-trial variation in subthalamic nucleus activity and variance in decision thresholds based on computational modeling analysis (Cohen and Cavanagh, 2011; Crowley et al., 2014; Rawls et al., 2020). Our findings indicate statistical significance between 200 and 400 ms, spanning frequencies of 1–10 Hz. This suggests that low-frequency activity could be a reliable marker for reward processing, providing more immediate and accurate understanding of the cognitive mechanisms underlying decision-making in situations with potential risks (Ratcliff and Frank, 2012; Cavanagh and Frank, 2014). Hence, more EEG signals can be utilized to examine neural mechanisms within RL models during dynamic decision-making and provide additional details about enduring brain-based characteristics.

Our study contributed novel advancements by widely employing BIT design without necessitating numeracy skills, as seen in BART (Lejuez et al., 2002). The variances between BIT and BART are rooted in the inflation method, transitioning from continuous clicks in BART to a single click in BIT. This modification in BIT allows for a more explicit revelation of decision-makers genuine preferences. Consequently, the choice variability reflects RA quantitatively, as each decision is uniformly assessed due to the consistent risk associated with each renewed balloon. Another crucial disparity is that while the burst probability signifies potential risk in both tasks, this crucial information cannot be acquired in BART due to its floating burst point, unlike in BIT, where risk ratios for each option are deliberately designed. Hence, the RL model in BIT facilitates the cognitive process of reward-driven choices and generates structured model parameters that index RS. These distinctive features of BIT contribute to the differentiation of reward responsivity from RS, where RA is directly influenced by behaviors impacting the most recent rewarded choice. At the same time, RS is associated with the learning pace in a reward-centric learning setting. Prior research utilizing questionnaires grounded in approach-avoidance personality theories has faced difficulties integrating perception/valuation sensitivity with motivation/action sensitivity, which impacts the observed behaviors (Corr and McNaughton, 2012; Nash and Knoch, 2016). The challenge arises in handling perception/valuation sensitivity and motivation/action sensitivity in self-report questionnaires. In BIT, money allows for easy manipulation through the presentation or omission of rewards, offering a significant advantage in modifying stimuli and altering states. Similar to concepts in previous quantitative frameworks related to self-valuation for rewards, RS scales the subjective value of potential choices guiding decision-making processes. In contrast, reward responsivity influences responses and behavioral control, known as RA. Consequently, BIT analyzed by RL models can provide direct indicators for RS and RA, which is suitable for neural analysis of RS.

Various significant limitations need consideration when interpreting the study results. The approach of treating SS as a trait with a total score, instead of analyzing subscale scores like experience-seeking and thrill-seeking, was chosen to facilitate comparisons with prior research and enhance the integrated understanding of SS. While utilizing total scores of SSS offers advantages, it also poses limitations. Future research should delve deeper into individual SS subscales and explore interaction effects between these subscales and other psychological constructs, such as RS, to enhance comprehension of the intricate dynamics of risk-taking. Additionally, while our study focused on quantifying RA using unknown but ordered risks within cognitive processes of dynamic decision-making, it is important to note that dynamic risk decision-making encompasses various contexts. As a result, the results from BIT experiment may not generalize to other forms of risk due to potential changes in the formation of risk aversion. Lastly, our outcomes relied on trial-by-trial variance instead of block-between-block analysis, presenting difficulties in merging ERP data and requiring artifact removal and noise cancellation across trials. Nonetheless, utilizing advanced techniques such as time-frequency analysis, source analysis, or even microstate analysis for ERP could provide resolutions and unveil additional evidence concerning the neural underpinnings associated with personality traits influencing risky decision-making.

## 6 Conclusion

In conclusion, our research findings have provided valuable insights into the influence of RS and SS on RA. The evidence suggests that individuals with high RS tend to take more risks and adapt their risk preferences based on their experiences. This underscores the importance of RS in shaping adjustments made for risky decisions in dynamic environments. Moreover, our study demonstrated that individuals with HSS showed reduced responsiveness to different types of feedback compared to those with LSS. This suggests SS may impact how individuals process and respond to feedback in decision-making contexts.

Overall, our findings contribute to a better understanding of how individual differences in RS and SS can shape decision-making processes, with potential implications for decision-making strategies and interventions in real-world settings. About solutions, our research highlights the importance of developing more sensitive behavioral measures for RA and neural presentation for sensation-seeking traits. Future research could benefit from employing advanced methods such as cognitive modeling and source analysis of ERP to investigate further the neural substrates related to personality traits influencing risky decision-making.

## Data Availability

The datasets presented in this study can be found in online repositories. The names of the repository/repositories and accession number(s) can be found in this article/Supplementary material.

## Footnotes

1
https://www.pstnet.com/
