# Supplementary material for: Sensation seeking and risk adjustment: the role of reward sensitivity in dynamic risky decisions
Source: Front Behav Neurosci. 2025 Feb 7;19:1492312. doi: 10.3389/fnbeh.2025.1492312 (PMC11842430; doi:10.3389/fnbeh.2025.1492312)

| **1.** Subscale interrelation | | | | | |
| --- | --- | --- | --- | --- | --- |
|  | | SSS1 | SSS2 | SSS3 | SSS4 |
| SSS1 | Pearson | 1 | .669^**^ | .522^**^ | .455^**^ |
|  | Sig. （two-tail） |  | .000 | .000 | .000 |
|  | Num | 80 | 80 | 80 | 80 |
| SSS2 | Pearson | .669^**^ | 1 | .567^**^ | .360^**^ |
|  | Sig. （two-tail） | .000 |  | .000 | .001 |
|  | Num | 80 | 80 | 80 | 80 |
| SSS3 | Pearson | .522^**^ | .567^**^ | 1 | .589^**^ |
|  | Sig. （two-tail） | .000 | .000 |  | .000 |
|  | Num | 80 | 80 | 80 | 80 |
| SSS4 | Pearson | .455^**^ | .360^**^ | .589^**^ | 1 |
|  | Sig.（two-tail） | .000 | .001 | .000 |  |
|  | Num | 80 | 80 | 80 | 80 |
| **. P< 0.01 | | | | | |

SSS1-thrill and adventure seeking, SSS2-boredom susceptibility, SSS3-experience seeking and SSS4-disinhibition


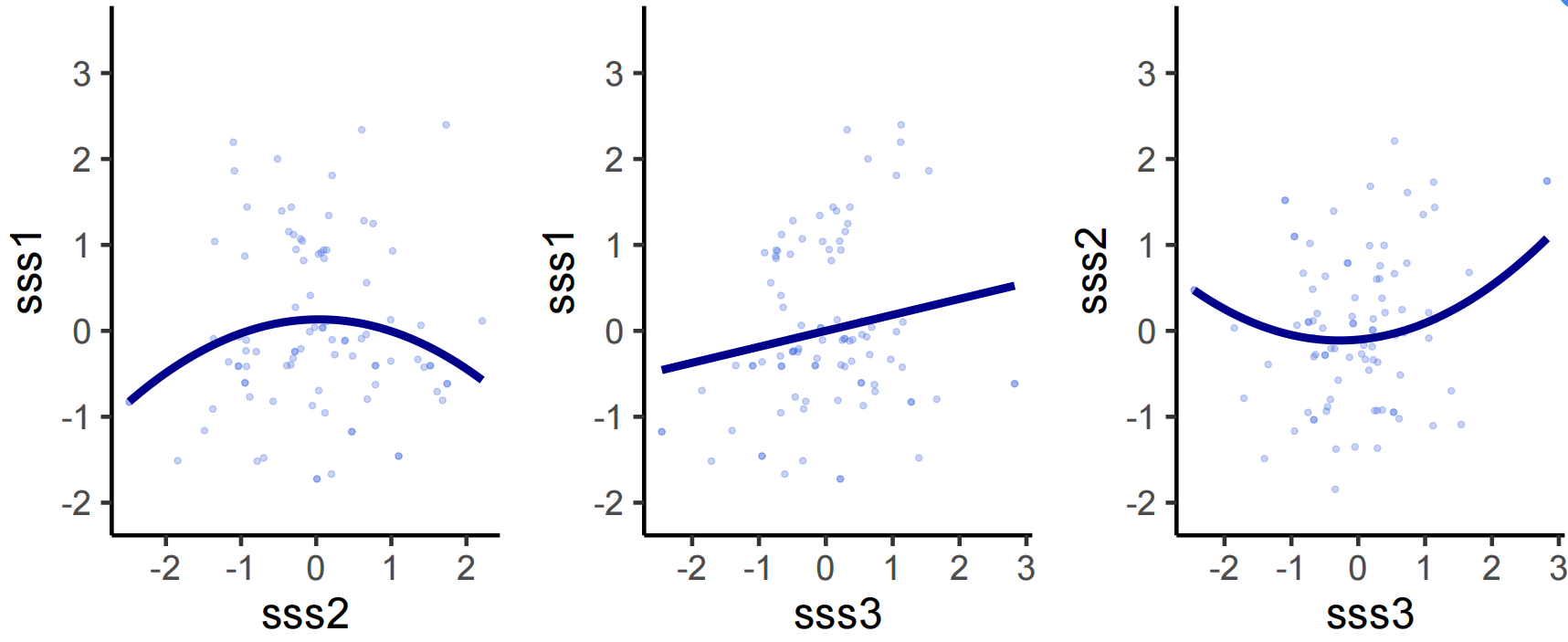


As the model fitting showed, SSS1 had a linear correlation with SSS3, while SSS2 had a quadruple correlation with SSS1 and SSS3.

2. The factor analysis

| **Total variance interpretation** | | | | | | |
| --- | --- | --- | --- | --- | --- | --- |
| ITEMS | Initial eigenvalue | | | Extract the sum of squares of the loads | | |
|  | SUM | PERCENTAGE | ACCUMULATE | TOTAL | VARIANCE | TOTAL |
| 1 | 8.336 | 20.839 | 20.839 | 8.336 | 20.839 | 20.839 |
| 2 | 5.654 | 14.136 | 34.975 | 5.654 | 14.136 | 34.975 |
| 3 | 3.133 | 7.831 | 42.806 | 3.133 | 7.831 | 42.806 |
| 4 | 2.408 | 6.020 | 48.826 | 2.408 | 6.020 | 48.826 |
| 5 | 2.129 | 5.322 | 54.148 | 2.129 | 5.322 | 54.148 |
| 6 | 1.630 | 4.074 | 58.223 | 1.630 | 4.074 | 58.223 |
| 7 | 1.439 | 3.598 | 61.820 | 1.439 | 3.598 | 61.820 |
| 8 | 1.361 | 3.401 | 65.222 | 1.361 | 3.401 | 65.222 |
| 9 | 1.328 | 3.320 | 68.542 | 1.328 | 3.320 | 68.542 |
| 10 | 1.139 | 2.848 | 71.390 | 1.139 | 2.848 | 71.390 |
| 11 | 1.093 | 2.733 | 74.124 | 1.093 | 2.733 | 74.124 |
| 12 | .924 | 2.310 | 76.434 |  |  |  |
| 13 | .900 | 2.251 | 78.685 |  |  |  |
| 14 | .823 | 2.057 | 80.742 |  |  |  |
| 15 | .747 | 1.868 | 82.610 |  |  |  |
| 16 | .688 | 1.720 | 84.331 |  |  |  |
| 17 | .633 | 1.584 | 85.914 |  |  |  |
| 18 | .565 | 1.413 | 87.327 |  |  |  |
| 19 | .529 | 1.323 | 88.650 |  |  |  |
| 20 | .522 | 1.305 | 89.955 |  |  |  |
| 21 | .479 | 1.198 | 91.153 |  |  |  |
| 22 | .438 | 1.095 | 92.249 |  |  |  |
| 23 | .368 | .920 | 93.168 |  |  |  |
| 24 | .341 | .853 | 94.021 |  |  |  |
| 25 | .321 | .803 | 94.824 |  |  |  |
| 26 | .283 | .708 | 95.532 |  |  |  |
| 27 | .263 | .658 | 96.190 |  |  |  |
| 28 | .243 | .607 | 96.796 |  |  |  |
| 29 | .208 | .520 | 97.317 |  |  |  |
| 30 | .175 | .438 | 97.755 |  |  |  |
| 31 | .169 | .422 | 98.177 |  |  |  |
| 32 | .141 | .353 | 98.529 |  |  |  |
| 33 | .126 | .316 | 98.845 |  |  |  |
| 34 | .103 | .257 | 99.103 |  |  |  |
| 35 | .099 | .248 | 99.351 |  |  |  |
| 36 | .079 | .198 | 99.549 |  |  |  |
| 37 | .065 | .163 | 99.712 |  |  |  |
| 38 | .045 | .114 | 99.826 |  |  |  |
| 39 | .038 | .094 | 99.920 |  |  |  |
| 40 | .032 | .080 | 100.000 |  |  |  |
|  | | | | | | |


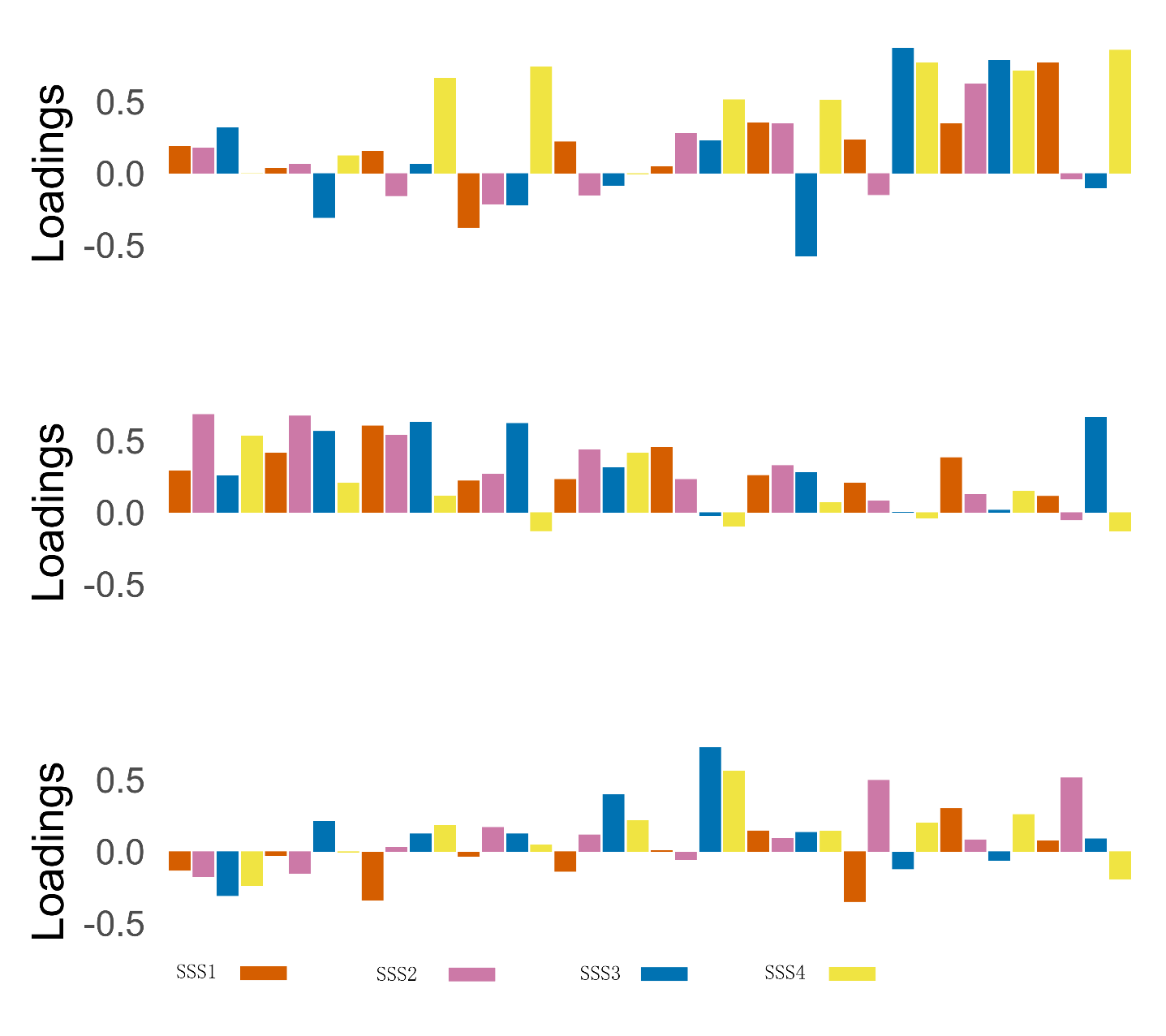


Factor 3

Factor 2

Factor 1

The major contributions were from two components, which accounted for the 20.84% and 14.14 variances respectively, while the rest of the components had similar contributions. Furthermore, we extracted the three factors and rotated them orthogonally. As the colorful figure showed, the items from the subscales were mixed in each factor. Therefore, it suggested the total score of subscales was better considering the four-factor structure was not fit in our samples as expected.

3. **The summary of each model with all samples was detailed as follows:**

**Inference for Stan model: RW_SM.**

4 chains, each with iter=2000; warmup=1000; thin=1;

post-warmup draws per chain=1000, total post-warmup draws=4000.

mean se_mean sd 2.5% 25%

mu_alpha_gr -1.28 0.00 0.10 -1.48 -1.35

mu_theta_gr 0.21 0.00 0.10 0.02 0.15

sigma_alpha_gr 0.54 0.00 0.09 0.39 0.48

sigma_theta_gr 0.39 0.00 0.10 0.23 0.33

alpha_ind[1] -0.65 0.01 0.56 -1.63 -1.02

alpha_ind[2] -0.36 0.01 0.62 -1.56 -0.77

alpha_ind[3] -0.89 0.01 0.44 -1.77 -1.18

alpha_ind[4] -0.23 0.01 0.59 -1.42 -0.62

alpha_ind[5] 1.44 0.01 0.51 0.41 1.11

alpha_ind[6] -0.44 0.01 0.40 -1.20 -0.68

alpha_ind[7] -0.08 0.01 0.33 -0.77 -0.29

alpha_ind[8] -0.21 0.01 0.44 -1.09 -0.51

alpha_ind[9] -0.55 0.01 0.51 -1.56 -0.88

alpha_ind[10] -0.37 0.01 0.59 -1.49 -0.76

alpha_ind[11] 0.46 0.01 0.39 -0.34 0.22

alpha_ind[12] -0.69 0.01 0.53 -1.73 -1.04

alpha_ind[13] -0.55 0.01 0.62 -1.71 -0.97

alpha_ind[14] -0.19 0.01 0.52 -1.19 -0.54

alpha_ind[15] 0.77 0.01 0.36 0.07 0.52

alpha_ind[16] 0.79 0.01 0.44 -0.08 0.50

alpha_ind[17] -0.19 0.01 0.33 -0.88 -0.40

alpha_ind[18] -0.72 0.01 0.55 -1.90 -1.05

alpha_ind[19] 0.75 0.02 0.79 -1.04 0.29

alpha_ind[20] 0.00 0.01 0.44 -0.85 -0.29

alpha_ind[21] -1.52 0.01 0.68 -2.94 -1.96

alpha_ind[22] 1.00 0.01 0.55 -0.06 0.64

alpha_ind[23] 1.88 0.01 0.43 1.06 1.59

alpha_ind[24] -0.08 0.01 0.64 -1.20 -0.52

alpha_ind[25] 0.95 0.01 0.35 0.31 0.71

alpha_ind[26] 0.54 0.05 1.45 -1.47 -0.58

alpha_ind[27] 0.24 0.01 0.46 -0.66 -0.06

alpha_ind[28] 2.37 0.01 0.56 1.34 1.98

alpha_ind[29] -0.44 0.01 0.50 -1.42 -0.78

alpha_ind[30] -0.13 0.01 0.38 -0.91 -0.37

alpha_ind[31] 1.39 0.01 0.43 0.60 1.09

alpha_ind[32] -0.40 0.01 0.44 -1.23 -0.68

alpha_ind[33] -0.42 0.01 0.44 -1.28 -0.71

alpha_ind[34] -0.57 0.01 0.43 -1.43 -0.86

alpha_ind[35] -0.72 0.01 0.39 -1.50 -0.98

alpha_ind[36] -0.33 0.01 0.49 -1.27 -0.65

alpha_ind[37] -1.09 0.01 0.66 -2.49 -1.49

alpha_ind[38] -0.97 0.01 0.62 -2.21 -1.35

alpha_ind[39] 0.01 0.01 0.31 -0.63 -0.19

alpha_ind[40] -0.06 0.01 0.59 -1.27 -0.44

alpha_ind[41] -0.01 0.01 0.55 -1.02 -0.37

alpha_ind[42] -1.00 0.01 0.62 -2.31 -1.39

alpha_ind[43] 1.25 0.01 0.43 0.39 0.97

theta_ind[1] -0.24 0.01 0.93 -2.03 -0.86

theta_ind[2] -0.61 0.01 0.87 -2.22 -1.22

theta_ind[3] 0.14 0.01 0.85 -1.49 -0.44

theta_ind[4] -0.39 0.01 0.89 -2.11 -0.99

theta_ind[5] -0.47 0.01 0.47 -1.45 -0.76

theta_ind[6] 1.43 0.01 0.82 -0.25 0.90

theta_ind[7] 0.73 0.01 0.46 -0.15 0.43

theta_ind[8] 0.21 0.01 0.64 -0.98 -0.21

theta_ind[9] -0.23 0.01 0.89 -1.95 -0.82

theta_ind[10] -0.27 0.01 0.89 -1.98 -0.88

theta_ind[11] -0.34 0.01 0.45 -1.28 -0.61

theta_ind[12] -0.15 0.01 0.92 -1.96 -0.78

theta_ind[13] -0.43 0.01 0.93 -2.19 -1.05

theta_ind[14] -0.10 0.01 0.84 -1.70 -0.68

theta_ind[15] -0.64 0.01 0.44 -1.60 -0.92

theta_ind[16] -0.84 0.01 0.49 -1.89 -1.13

theta_ind[17] 1.70 0.01 0.50 0.80 1.35

theta_ind[18] -0.06 0.01 0.96 -1.90 -0.70

theta_ind[19] -1.48 0.02 0.71 -2.73 -1.94

theta_ind[20] 0.95 0.01 0.61 -0.20 0.54

theta_ind[21] -0.56 0.01 1.01 -2.56 -1.24

theta_ind[22] 0.10 0.01 0.50 -0.89 -0.21

theta_ind[23] 0.12 0.01 0.38 -0.63 -0.12

theta_ind[24] -0.26 0.01 0.83 -1.78 -0.83

theta_ind[25] 0.59 0.01 0.36 -0.10 0.35

theta_ind[26] -0.97 0.03 1.03 -2.69 -1.75

theta_ind[27] -0.02 0.01 0.60 -1.26 -0.38

theta_ind[28] -0.34 0.01 0.41 -1.17 -0.61

theta_ind[29] -0.13 0.01 0.87 -1.78 -0.72

theta_ind[30] 0.25 0.01 0.49 -0.70 -0.06

theta_ind[31] -0.14 0.01 0.41 -1.01 -0.40

theta_ind[32] 1.47 0.01 0.83 -0.19 0.92

theta_ind[33] 0.21 0.01 0.72 -1.15 -0.29

theta_ind[34] 0.50 0.01 0.74 -0.95 0.00

theta_ind[35] 0.95 0.01 0.74 -0.53 0.45

theta_ind[36] 0.12 0.01 0.87 -1.59 -0.45

theta_ind[37] -0.43 0.01 0.96 -2.25 -1.09

theta_ind[38] -0.48 0.01 0.97 -2.42 -1.12

theta_ind[39] 0.76 0.01 0.40 0.00 0.50

theta_ind[40] -0.75 0.01 0.75 -2.16 -1.25

theta_ind[41] 0.28 0.01 0.81 -1.23 -0.27

theta_ind[42] -0.38 0.01 0.96 -2.22 -1.04

theta_ind[43] 0.43 0.01 0.39 -0.33 0.18

mu_alpha_gr_tr 0.10 0.00 0.02 0.07 0.09

mu_theta_gr_tr 1.24 0.00 0.12 1.02 1.17

alpha_ind_tr[1] 0.06 0.00 0.04 0.02 0.03

alpha_ind_tr[2] 0.08 0.00 0.05 0.02 0.05

alpha_ind_tr[3] 0.04 0.00 0.02 0.01 0.03

alpha_ind_tr[4] 0.09 0.00 0.05 0.02 0.05

alpha_ind_tr[5] 0.31 0.00 0.08 0.15 0.25

alpha_ind_tr[6] 0.07 0.00 0.03 0.03 0.05

alpha_ind_tr[7] 0.10 0.00 0.02 0.05 0.08

alpha_ind_tr[8] 0.09 0.00 0.03 0.03 0.06

50% 75% 97.5% n_eff Rhat

mu_alpha_gr -1.28 -1.21 -1.08 1074 1.00

mu_theta_gr 0.22 0.28 0.40 1258 1.00

sigma_alpha_gr 0.53 0.59 0.74 1020 1.00

sigma_theta_gr 0.39 0.45 0.60 918 1.00

alpha_ind[1] -0.70 -0.35 0.68 2814 1.00

alpha_ind[2] -0.38 0.06 0.88 3957 1.00

alpha_ind[3] -0.88 -0.59 -0.07 2990 1.00

alpha_ind[4] -0.22 0.16 0.91 3996 1.00

alpha_ind[5] 1.45 1.77 2.45 3591 1.00

alpha_ind[6] -0.44 -0.18 0.34 2282 1.00

alpha_ind[7] -0.07 0.14 0.54 2216 1.00

alpha_ind[8] -0.20 0.10 0.63 2945 1.00

alpha_ind[9] -0.55 -0.22 0.46 4046 1.00

alpha_ind[10] -0.37 0.00 0.80 4386 1.00

alpha_ind[11] 0.46 0.71 1.20 2919 1.00

alpha_ind[12] -0.69 -0.35 0.36 3799 1.00

alpha_ind[13] -0.56 -0.17 0.74 3191 1.00

alpha_ind[14] -0.20 0.15 0.84 3709 1.00

alpha_ind[15] 0.76 1.00 1.51 2448 1.00

alpha_ind[16] 0.79 1.07 1.66 3488 1.00

alpha_ind[17] -0.18 0.03 0.40 1894 1.00

alpha_ind[18] -0.70 -0.37 0.30 3937 1.00

alpha_ind[19] 0.88 1.31 2.04 1873 1.00

alpha_ind[20] 0.00 0.28 0.89 2559 1.00

alpha_ind[21] -1.50 -1.06 -0.23 5103 1.00

alpha_ind[22] 0.98 1.37 2.12 4034 1.00

alpha_ind[23] 1.87 2.16 2.76 1590 1.00

alpha_ind[24] -0.13 0.29 1.36 3970 1.00

alpha_ind[25] 0.94 1.19 1.67 1773 1.00

alpha_ind[26] 0.05 1.87 3.35 718 1.01

alpha_ind[27] 0.24 0.53 1.18 4126 1.00

alpha_ind[28] 2.36 2.73 3.53 3261 1.00

alpha_ind[29] -0.44 -0.11 0.55 3796 1.00

alpha_ind[30] -0.12 0.12 0.58 2791 1.00

alpha_ind[31] 1.38 1.68 2.28 2442 1.00

alpha_ind[32] -0.41 -0.11 0.50 2159 1.00

alpha_ind[33] -0.41 -0.12 0.45 2973 1.00

alpha_ind[34] -0.57 -0.28 0.26 3374 1.00

alpha_ind[35] -0.71 -0.45 0.01 2447 1.00

alpha_ind[36] -0.34 -0.02 0.65 3844 1.00

alpha_ind[37] -1.06 -0.65 0.11 4212 1.00

alpha_ind[38] -0.98 -0.56 0.25 3949 1.00

alpha_ind[39] 0.01 0.22 0.60 2167 1.00

alpha_ind[40] -0.03 0.35 1.06 3704 1.00

alpha_ind[41] -0.04 0.33 1.17 3512 1.00

alpha_ind[42] -0.97 -0.58 0.16 4324 1.00

alpha_ind[43] 1.25 1.53 2.07 2416 1.00

theta_ind[1] -0.26 0.38 1.62 3864 1.00

theta_ind[2] -0.64 -0.05 1.16 3862 1.00

theta_ind[3] 0.13 0.70 1.85 4558 1.00

theta_ind[4] -0.42 0.19 1.48 4863 1.00

theta_ind[5] -0.46 -0.16 0.41 3344 1.00

theta_ind[6] 1.46 1.99 2.92 4218 1.00

theta_ind[7] 0.71 1.03 1.70 3177 1.00

theta_ind[8] 0.17 0.62 1.49 3285 1.00

theta_ind[9] -0.23 0.38 1.52 5564 1.00

theta_ind[10] -0.30 0.34 1.53 4794 1.00

theta_ind[11] -0.31 -0.04 0.49 2987 1.00

theta_ind[12] -0.15 0.46 1.64 5271 1.00

theta_ind[13] -0.44 0.19 1.44 5603 1.00

theta_ind[14] -0.11 0.44 1.62 4309 1.00

theta_ind[15] -0.62 -0.33 0.16 2436 1.00

theta_ind[16] -0.81 -0.51 0.02 2392 1.00

theta_ind[17] 1.66 2.01 2.75 2134 1.00

theta_ind[18] -0.03 0.59 1.74 7446 1.00

theta_ind[19] -1.53 -1.10 0.18 2187 1.00

theta_ind[20] 0.93 1.33 2.20 4469 1.00

theta_ind[21] -0.57 0.14 1.42 5630 1.00

theta_ind[22] 0.11 0.42 1.08 3608 1.00

theta_ind[23] 0.13 0.37 0.84 2550 1.00

theta_ind[24] -0.28 0.30 1.47 4869 1.00

theta_ind[25] 0.57 0.82 1.34 2335 1.00

theta_ind[26] -1.09 -0.26 1.17 1388 1.00

theta_ind[27] -0.02 0.36 1.16 4139 1.00

theta_ind[28] -0.33 -0.06 0.40 2510 1.00

theta_ind[29] -0.14 0.45 1.63 5479 1.00

theta_ind[30] 0.25 0.55 1.23 3380 1.00

theta_ind[31] -0.12 0.15 0.60 2970 1.00

theta_ind[32] 1.49 2.03 3.07 4516 1.00

theta_ind[33] 0.20 0.68 1.70 4686 1.00

theta_ind[34] 0.49 0.97 1.98 5109 1.00

theta_ind[35] 0.94 1.43 2.45 5022 1.00

theta_ind[36] 0.12 0.69 1.82 4981 1.00

theta_ind[37] -0.45 0.20 1.48 5764 1.00

theta_ind[38] -0.48 0.17 1.48 5507 1.00

theta_ind[39] 0.76 1.02 1.56 2683 1.00

theta_ind[40] -0.78 -0.31 0.87 3157 1.00

theta_ind[41] 0.26 0.81 1.92 4601 1.00

theta_ind[42] -0.38 0.26 1.56 5299 1.00

theta_ind[43] 0.43 0.69 1.22 2420 1.00

mu_alpha_gr_tr 0.10 0.11 0.14 1068 1.00

mu_theta_gr_tr 1.24 1.32 1.49 1249 1.00

alpha_ind_tr[1] 0.05 0.07 0.18 2399 1.00

alpha_ind_tr[2] 0.07 0.11 0.21 3364 1.00

alpha_ind_tr[3] 0.04 0.05 0.09 4194 1.00

alpha_ind_tr[4] 0.08 0.12 0.22 3881 1.00

alpha_ind_tr[5] 0.31 0.36 0.48 4871 1.00

alpha_ind_tr[6] 0.07 0.08 0.13 3583 1.00

alpha_ind_tr[7] 0.09 0.11 0.14 5819 1.00

alpha_ind_tr[8] 0.08 0.11 0.17 4427 1.00

[到达getOption("max.print") -- 略过2720行]]

Samples were drawn using NUTS(diag_e) at Sat Oct 02 19:30:49 2021.

For each parameter, n_eff is a crude measure of effective sample size,

and Rhat is the potential scale reduction factor on split chains (at

convergence, Rhat=1).

**Inference for Stan model: KFL_SM.**

4 chains, each with iter=2000; warmup=1000; thin=1;

post-warmup draws per chain=1000, total post-warmup draws=4000.

mean se_mean sd 2.5% 25%

mu_varm_gr -2.00 0.03 0.88 -3.57 -2.61

mu_vari_gr 2.12 0.03 0.74 0.71 1.60

mu_theta_gr 0.25 0.00 0.11 0.02 0.18

mu_eta_gr -0.80 0.01 0.24 -1.21 -0.97

sigma_varm_gr 2.10 0.05 0.98 0.16 1.40

sigma_vari_gr 1.56 0.04 0.77 0.19 1.01

sigma_theta_gr 0.46 0.00 0.11 0.27 0.39

sigma_eta_gr 0.79 0.01 0.18 0.50 0.66

varm_ind[1] -0.18 0.01 1.01 -2.06 -0.89

varm_ind[2] -0.37 0.01 1.00 -2.17 -1.04

varm_ind[3] -0.22 0.02 1.04 -2.18 -0.91

varm_ind[4] 0.08 0.01 0.87 -1.67 -0.48

varm_ind[5] -0.15 0.01 0.96 -1.98 -0.80

varm_ind[6] -0.22 0.01 0.71 -1.61 -0.63

varm_ind[7] -0.62 0.02 0.89 -2.31 -1.19

varm_ind[8] -0.09 0.01 0.93 -1.95 -0.72

varm_ind[9] -0.09 0.01 1.01 -2.01 -0.80

varm_ind[10] -0.08 0.01 1.00 -2.01 -0.73

varm_ind[11] 0.02 0.01 1.01 -1.95 -0.66

varm_ind[12] -0.42 0.02 1.03 -2.25 -1.13

varm_ind[13] 0.00 0.01 0.94 -1.88 -0.59

varm_ind[14] 0.00 0.01 0.95 -1.88 -0.62

varm_ind[15] 0.36 0.01 0.68 -0.89 -0.08

varm_ind[16] -0.65 0.01 0.87 -2.26 -1.20

varm_ind[17] -0.28 0.01 0.88 -2.03 -0.83

varm_ind[18] -0.32 0.01 1.00 -2.20 -1.00

varm_ind[19] 0.41 0.01 0.91 -1.52 -0.13

varm_ind[20] -0.23 0.01 1.02 -2.12 -0.93

varm_ind[21] -0.24 0.01 1.00 -2.15 -0.92

varm_ind[22] 0.30 0.01 0.99 -1.73 -0.31

varm_ind[23] 0.79 0.02 0.89 -1.23 0.32

varm_ind[24] -0.43 0.01 0.96 -2.26 -1.07

varm_ind[25] -0.32 0.02 0.79 -2.01 -0.79

varm_ind[26] 0.61 0.04 1.15 -1.61 -0.22

varm_ind[27] -0.18 0.01 0.93 -2.00 -0.78

varm_ind[28] 1.32 0.04 1.11 -1.32 0.77

varm_ind[29] -0.52 0.02 0.97 -2.34 -1.16

varm_ind[30] -0.52 0.02 1.00 -2.31 -1.19

varm_ind[31] 0.59 0.01 0.87 -1.32 0.10

varm_ind[32] -0.07 0.02 0.95 -1.93 -0.73

varm_ind[33] -0.32 0.02 0.98 -2.10 -0.98

varm_ind[34] 0.00 0.01 1.05 -1.99 -0.73

varm_ind[35] -0.11 0.02 1.02 -2.06 -0.81

varm_ind[36] -0.32 0.01 0.97 -2.10 -0.96

varm_ind[37] -0.16 0.01 0.89 -1.88 -0.75

varm_ind[38] -0.08 0.01 0.92 -1.96 -0.67

varm_ind[39] -0.86 0.02 0.82 -2.47 -1.39

varm_ind[40] -0.05 0.01 0.99 -1.99 -0.69

varm_ind[41] -0.75 0.02 0.92 -2.42 -1.36

varm_ind[42] -0.20 0.01 0.98 -2.09 -0.85

varm_ind[43] 0.55 0.02 1.07 -1.63 -0.16

vari_ind[1] 0.00 0.01 0.93 -1.72 -0.64

vari_ind[2] 0.07 0.01 0.84 -1.57 -0.48

vari_ind[3] 0.05 0.01 0.97 -1.82 -0.62

vari_ind[4] -0.40 0.01 0.98 -2.18 -1.07

vari_ind[5] 0.57 0.01 0.81 -1.09 0.06

vari_ind[6] -1.36 0.03 0.96 -3.01 -1.98

vari_ind[7] 0.04 0.01 0.81 -1.48 -0.49

vari_ind[8] -0.07 0.01 0.92 -1.83 -0.70

vari_ind[9] -0.01 0.01 0.93 -1.81 -0.63

vari_ind[10] -0.02 0.01 0.94 -1.84 -0.67

vari_ind[11] 0.26 0.01 0.91 -1.54 -0.32

vari_ind[12] -0.09 0.01 0.92 -1.87 -0.71

vari_ind[13] -0.30 0.01 1.04 -2.17 -1.01

vari_ind[14] -0.03 0.01 1.02 -2.00 -0.74

vari_ind[15] -0.69 0.02 1.01 -2.44 -1.39

vari_ind[16] 0.40 0.01 0.76 -1.09 -0.07

vari_ind[17] -0.32 0.01 0.86 -1.89 -0.88

vari_ind[18] -0.13 0.01 0.93 -1.90 -0.75

vari_ind[19] 0.04 0.01 1.09 -2.08 -0.68

vari_ind[20] 0.22 0.01 0.89 -1.41 -0.40

vari_ind[21] -0.29 0.01 1.03 -2.25 -1.00

vari_ind[22] 0.16 0.01 0.96 -1.69 -0.48

vari_ind[23] 0.72 0.02 0.99 -1.28 0.11

vari_ind[24] -0.20 0.01 0.88 -1.81 -0.80

vari_ind[25] 0.52 0.02 0.72 -0.83 0.06

vari_ind[26] 0.38 0.05 1.25 -1.96 -0.54

vari_ind[27] 0.06 0.01 0.84 -1.52 -0.51

vari_ind[28] 0.46 0.03 1.09 -1.77 -0.30

vari_ind[29] 0.05 0.01 0.82 -1.51 -0.47

vari_ind[30] 0.32 0.01 0.81 -1.28 -0.22

vari_ind[31] 0.23 0.02 0.97 -1.63 -0.40

vari_ind[32] -0.67 0.01 0.92 -2.32 -1.29

vari_ind[33] 0.13 0.01 0.91 -1.57 -0.50

vari_ind[34] 0.09 0.01 0.98 -1.75 -0.59

vari_ind[35] 0.16 0.01 0.96 -1.75 -0.49

vari_ind[36] -0.25 0.01 0.92 -1.94 -0.86

vari_ind[37] -0.52 0.02 1.08 -2.49 -1.32

vari_ind[38] -0.47 0.02 1.09 -2.49 -1.24

vari_ind[39] 0.65 0.01 0.70 -0.70 0.20

vari_ind[40] 0.02 0.01 0.96 -1.85 -0.65

vari_ind[41] -0.07 0.01 0.74 -1.47 -0.55

vari_ind[42] -0.20 0.01 1.00 -2.05 -0.90

vari_ind[43] 0.79 0.03 1.14 -1.44 -0.04

theta_ind[1] -0.22 0.01 0.92 -1.88 -0.87

theta_ind[2] -0.83 0.01 0.84 -2.33 -1.40

theta_ind[3] 0.16 0.01 0.89 -1.57 -0.42

theta_ind[4] -0.43 0.01 0.82 -1.98 -0.98

theta_ind[5] -0.45 0.01 0.43 -1.32 -0.70

theta_ind[6] 1.88 0.01 0.67 0.46 1.48

50% 75% 97.5% n_eff Rhat

mu_varm_gr -2.04 -1.42 -0.19 684 1.00

mu_vari_gr 2.12 2.61 3.60 821 1.00

mu_theta_gr 0.25 0.32 0.46 1054 1.00

mu_eta_gr -0.82 -0.65 -0.27 595 1.01

sigma_varm_gr 2.19 2.80 3.88 345 1.01

sigma_vari_gr 1.49 2.07 3.13 367 1.01

sigma_theta_gr 0.45 0.53 0.71 568 1.01

sigma_eta_gr 0.77 0.90 1.21 1118 1.01

varm_ind[1] -0.25 0.50 1.88 6448 1.00

varm_ind[2] -0.44 0.24 1.79 5118 1.00

varm_ind[3] -0.27 0.46 1.92 4767 1.00

varm_ind[4] 0.07 0.63 1.78 6120 1.00

varm_ind[5] -0.17 0.45 1.85 4476 1.00

varm_ind[6] -0.23 0.17 1.34 3450 1.00

varm_ind[7] -0.64 -0.07 1.21 2719 1.00

varm_ind[8] -0.12 0.54 1.70 5923 1.00

varm_ind[9] -0.09 0.60 1.89 6262 1.00

varm_ind[10] -0.10 0.56 1.99 6931 1.00

varm_ind[11] 0.00 0.70 1.96 5220 1.00

varm_ind[12] -0.48 0.24 1.73 4545 1.00

varm_ind[13] 0.00 0.59 1.89 7793 1.00

varm_ind[14] 0.01 0.61 1.85 6740 1.00

varm_ind[15] 0.29 0.74 1.85 3668 1.00

varm_ind[16] -0.69 -0.15 1.22 3390 1.00

varm_ind[17] -0.27 0.27 1.53 4138 1.00

varm_ind[18] -0.37 0.31 1.77 4665 1.00

varm_ind[19] 0.44 0.99 2.17 4310 1.00

varm_ind[20] -0.29 0.45 1.81 5037 1.00

varm_ind[21] -0.29 0.40 1.82 5948 1.00

varm_ind[22] 0.36 0.96 2.16 5538 1.00

varm_ind[23] 0.83 1.34 2.42 2568 1.00

varm_ind[24] -0.47 0.17 1.61 4970 1.00

varm_ind[25] -0.29 0.16 1.28 2645 1.00

varm_ind[26] 0.64 1.49 2.67 819 1.00

varm_ind[27] -0.20 0.40 1.76 5599 1.00

varm_ind[28] 1.50 2.07 3.15 704 1.01

varm_ind[29] -0.58 0.09 1.51 3961 1.00

varm_ind[30] -0.61 0.08 1.65 3123 1.00

varm_ind[31] 0.63 1.14 2.26 3761 1.00

varm_ind[32] -0.07 0.60 1.75 3887 1.00

varm_ind[33] -0.40 0.34 1.74 4116 1.00

varm_ind[34] -0.01 0.72 2.06 6239 1.00

varm_ind[35] -0.15 0.58 1.89 3691 1.00

varm_ind[36] -0.36 0.32 1.65 5100 1.00

varm_ind[37] -0.18 0.42 1.59 6302 1.00

varm_ind[38] -0.08 0.52 1.74 5932 1.00

varm_ind[39] -0.86 -0.36 0.79 1842 1.00

varm_ind[40] -0.06 0.61 1.89 6618 1.00

varm_ind[41] -0.80 -0.24 1.27 2652 1.00

varm_ind[42] -0.23 0.41 1.84 6174 1.00

varm_ind[43] 0.58 1.33 2.49 2155 1.00

vari_ind[1] -0.04 0.59 1.92 6595 1.00

vari_ind[2] 0.06 0.60 1.78 6525 1.00

vari_ind[3] 0.03 0.69 1.97 5083 1.00

vari_ind[4] -0.47 0.24 1.64 4553 1.00

vari_ind[5] 0.57 1.09 2.14 3445 1.00

vari_ind[6] -1.49 -0.91 0.94 1283 1.00

vari_ind[7] 0.02 0.55 1.68 4864 1.00

vari_ind[8] -0.09 0.54 1.79 4596 1.00

vari_ind[9] -0.03 0.60 1.92 6437 1.00

vari_ind[10] -0.03 0.60 1.85 6321 1.00

vari_ind[11] 0.25 0.85 2.05 5611 1.00

vari_ind[12] -0.15 0.48 1.84 6296 1.00

vari_ind[13] -0.34 0.39 1.82 5218 1.00

vari_ind[14] -0.04 0.63 2.01 6601 1.00

vari_ind[15] -0.78 -0.08 1.53 2479 1.00

vari_ind[16] 0.38 0.87 1.93 3685 1.00

vari_ind[17] -0.36 0.21 1.56 4175 1.00

vari_ind[18] -0.16 0.48 1.76 5978 1.00

vari_ind[19] 0.03 0.80 2.11 5360 1.00

vari_ind[20] 0.18 0.80 2.03 5908 1.00

vari_ind[21] -0.33 0.38 1.81 5623 1.00

vari_ind[22] 0.12 0.79 2.10 5564 1.00

vari_ind[23] 0.70 1.32 2.78 1653 1.00

vari_ind[24] -0.25 0.37 1.63 5788 1.00

vari_ind[25] 0.48 0.97 2.01 2093 1.00

vari_ind[26] 0.34 1.30 2.77 726 1.01

vari_ind[27] 0.01 0.59 1.78 5578 1.00

vari_ind[28] 0.52 1.23 2.45 1183 1.00

vari_ind[29] 0.03 0.55 1.77 6551 1.00

vari_ind[30] 0.30 0.84 1.96 5838 1.00

vari_ind[31] 0.18 0.85 2.32 2933 1.00

vari_ind[32] -0.75 -0.11 1.32 3765 1.00

vari_ind[33] 0.08 0.72 1.99 5425 1.00

vari_ind[34] 0.07 0.76 2.07 5269 1.00

vari_ind[35] 0.13 0.82 2.06 4499 1.00

vari_ind[36] -0.30 0.35 1.69 5884 1.00

vari_ind[37] -0.59 0.23 1.69 3374 1.00

vari_ind[38] -0.53 0.29 1.70 3702 1.00

vari_ind[39] 0.64 1.09 2.10 2353 1.00

vari_ind[40] 0.01 0.66 1.91 5912 1.00

vari_ind[41] -0.10 0.39 1.50 5734 1.00

vari_ind[42] -0.24 0.47 1.78 5815 1.00

vari_ind[43] 0.84 1.65 2.85 1105 1.00

theta_ind[1] -0.24 0.40 1.62 5248 1.00

theta_ind[2] -0.89 -0.32 1.01 4087 1.00

theta_ind[3] 0.14 0.75 1.94 5776 1.00

theta_ind[4] -0.46 0.08 1.33 4804 1.00

theta_ind[5] -0.43 -0.15 0.33 1835 1.00

theta_ind[6] 1.91 2.31 3.12 2976 1.00

[到达getOption("max.print") -- 略过2925行]]

Samples were drawn using NUTS(diag_e) at Sat Oct 02 20:47:15 2021.

For each parameter, n_eff is a crude measure of effective sample size,

, and Rhat is the potential scale reduction factor on split chains (at

convergence, Rhat=1).

Here, we extracted the results for the posterior distributions shown in figure 1.(1) and 1.(2).


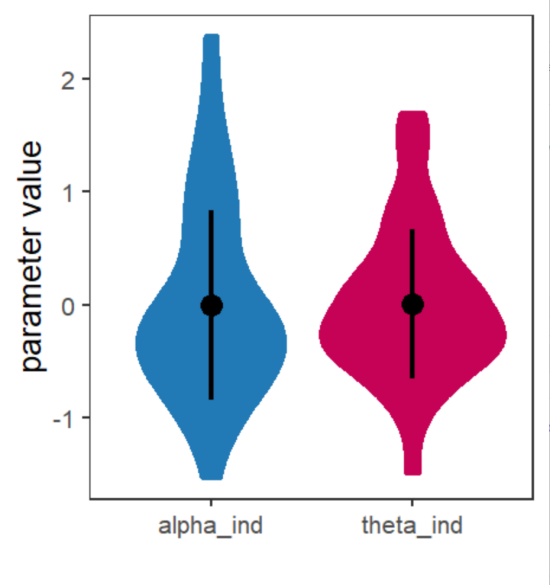
(1)RL model of two parameters (2) KL model of four parameters


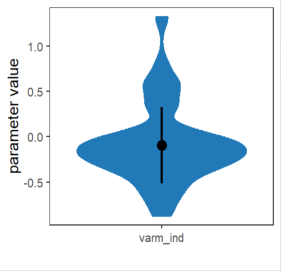

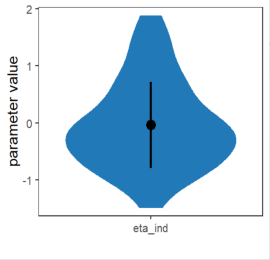

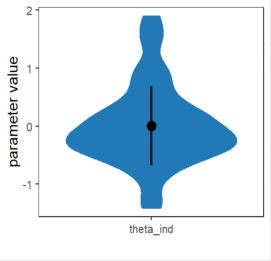

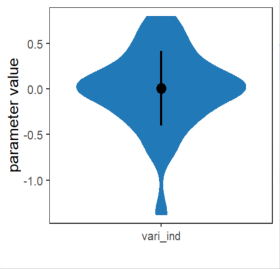


Figure 1. Posterior distributions for each parameter at the individual level.

Results in Figure 2 showed that the posterior predictions of the two models captured the key pattern similarly, however, they all did not capture the behavior of choosing 50% as expected as the model varied more stably than the actual behavior. Hence, there could be more potential factors uncovered in the two models.


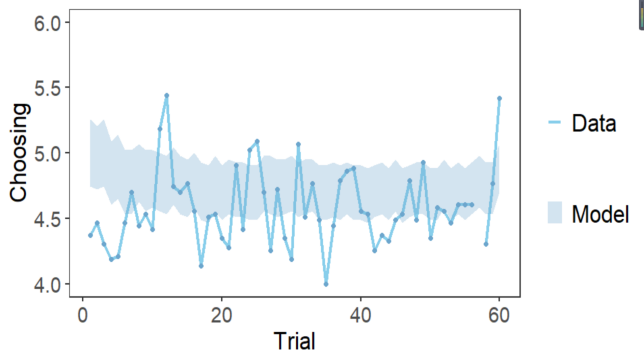

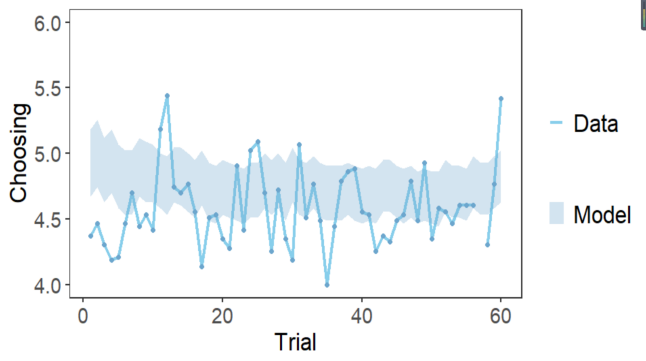


Figure 2 The posterior predictive simulations versus actual average choices

4. the Supplied files for BML

Study 1 : a null model as a reference


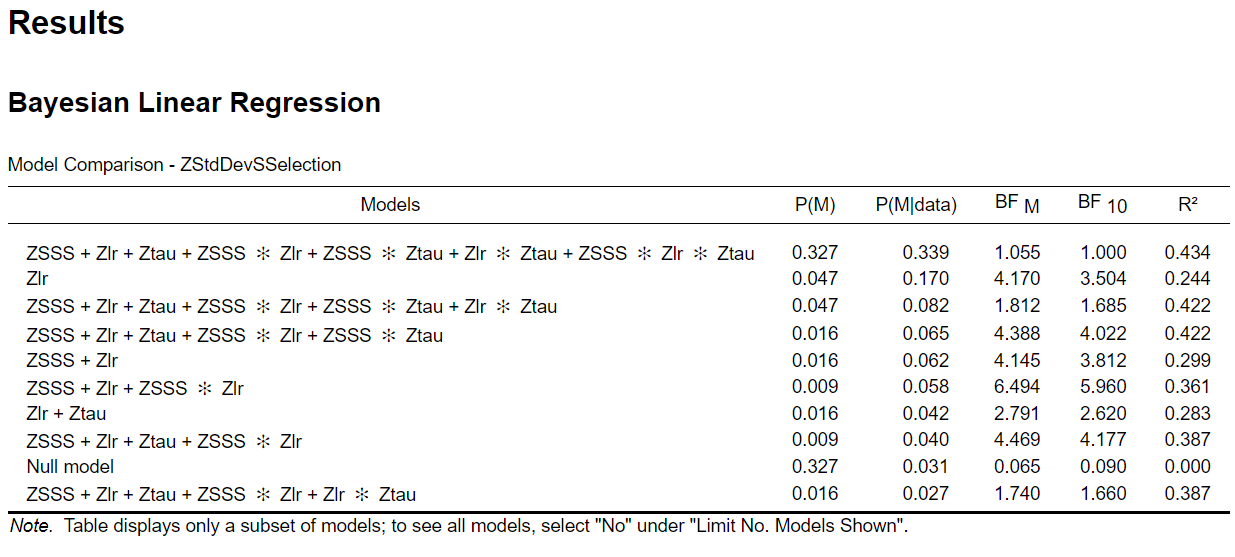


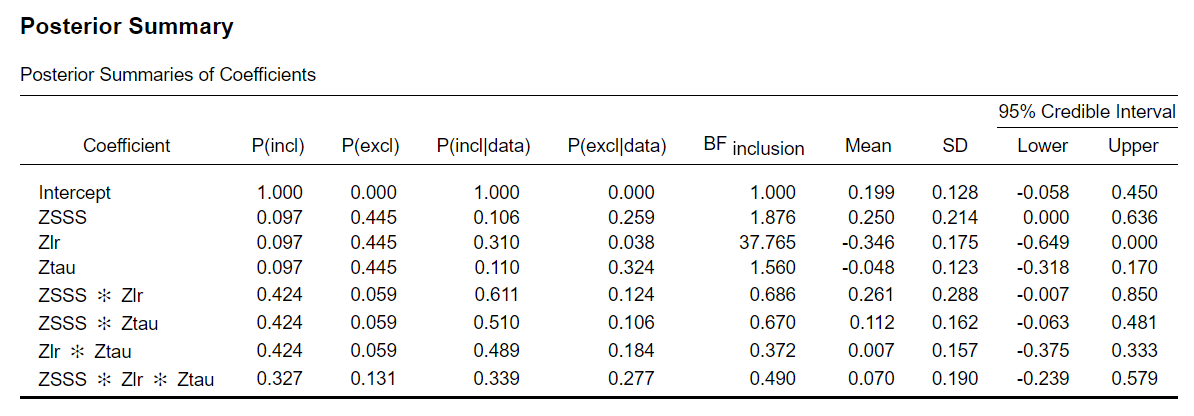


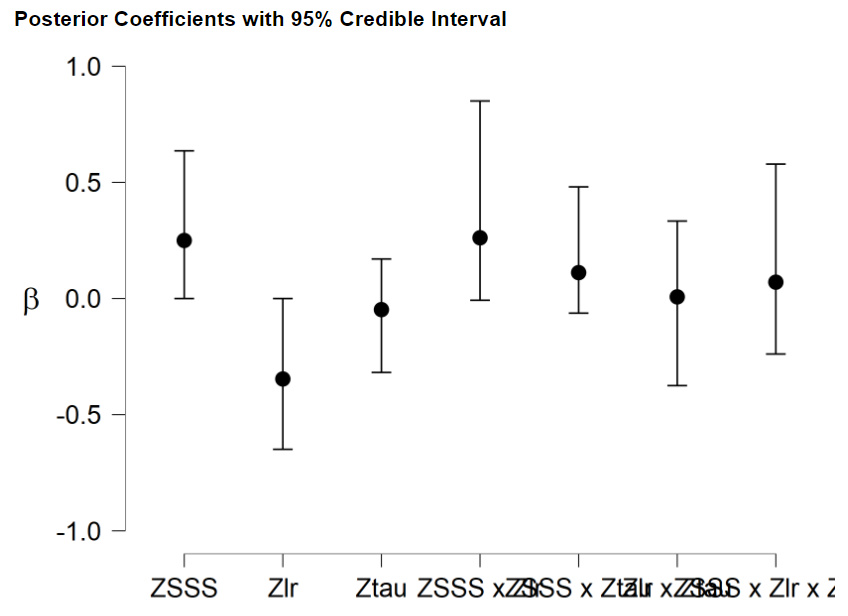

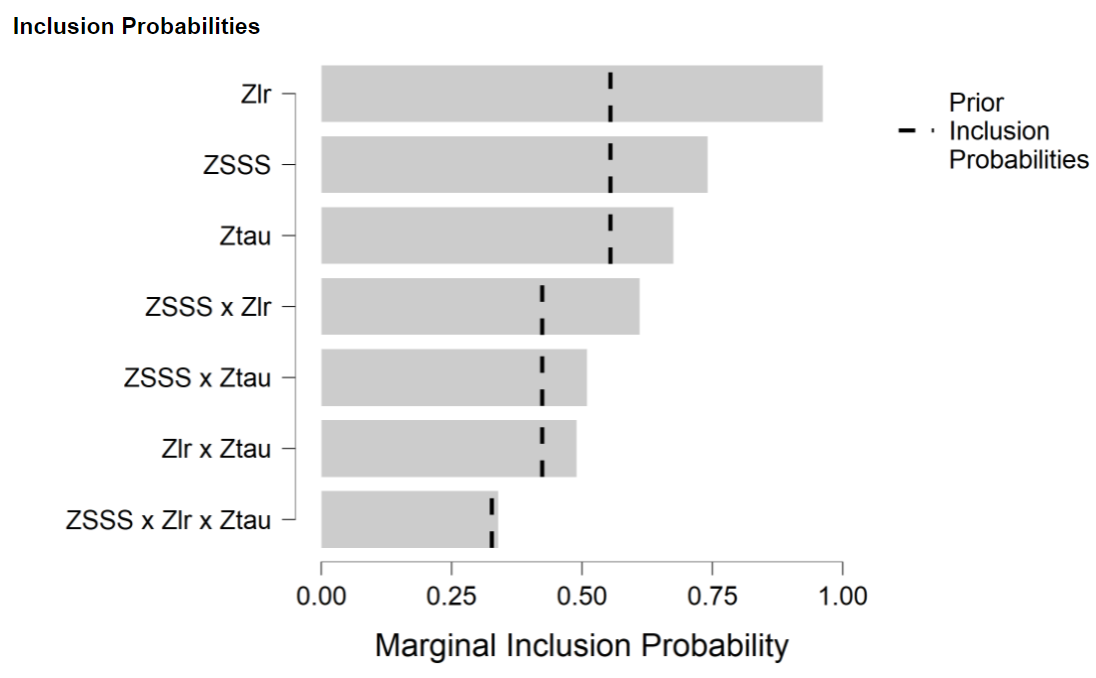


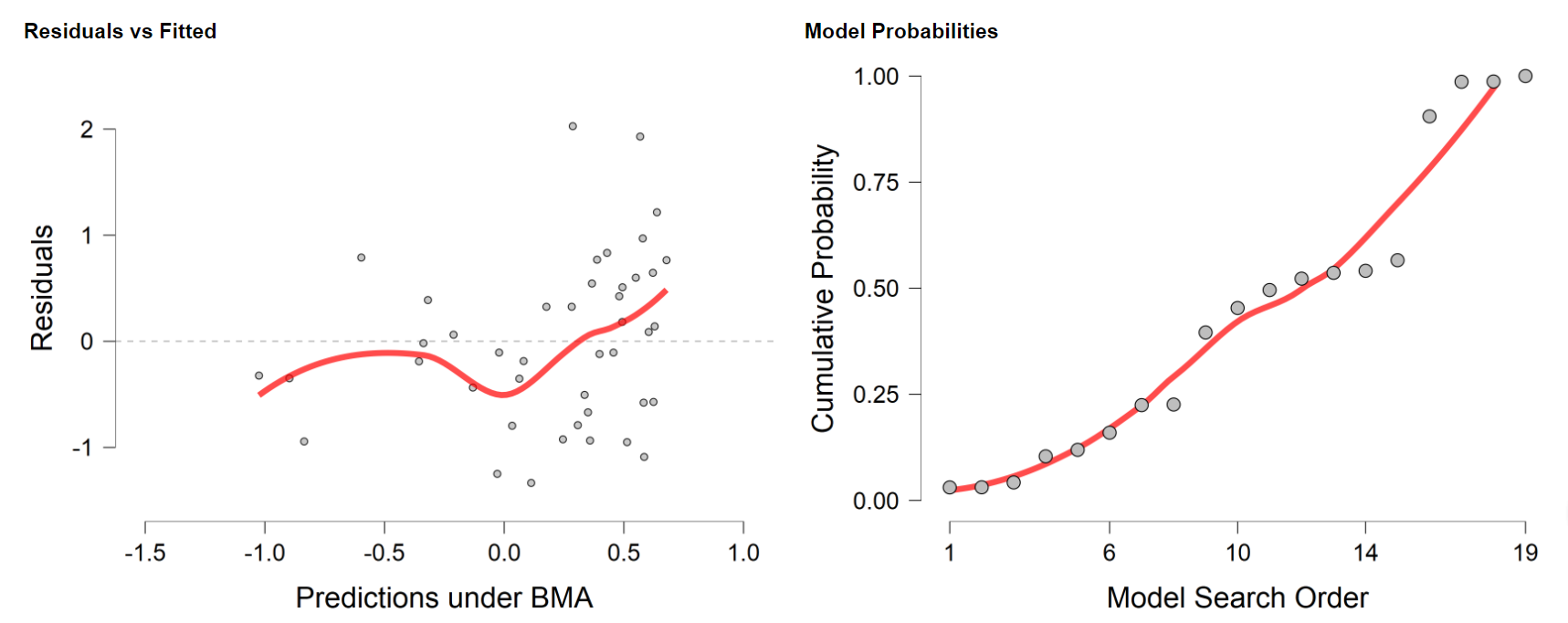


Study 2.1: the null model included lr and tau as a reference


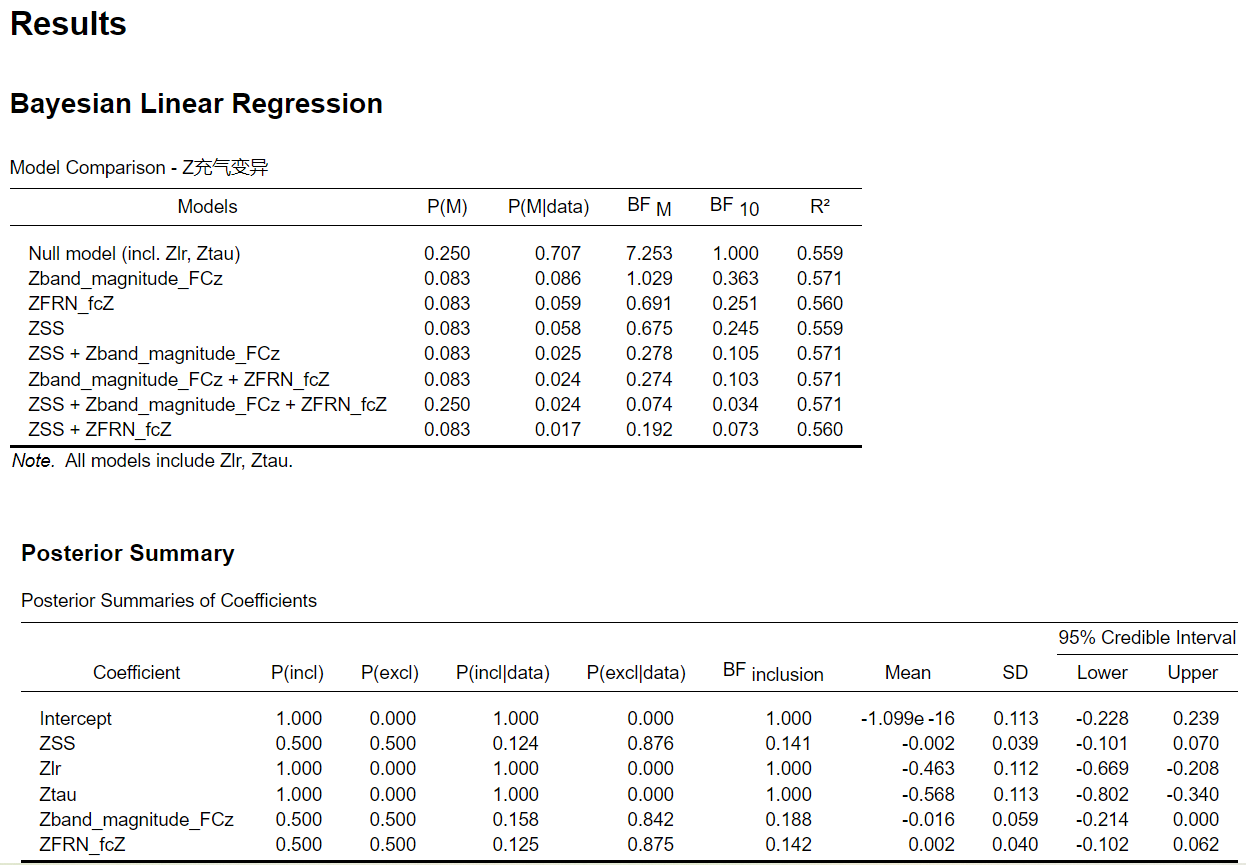


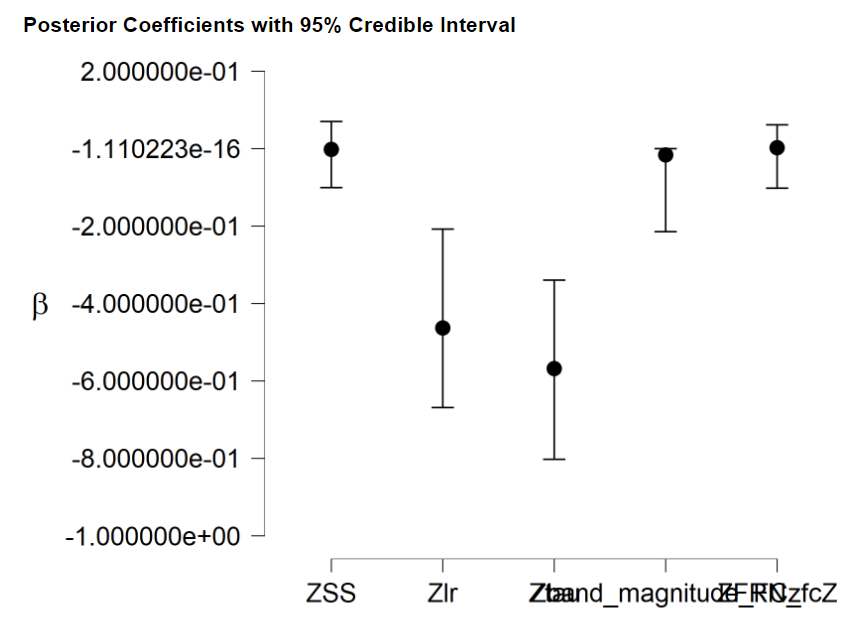

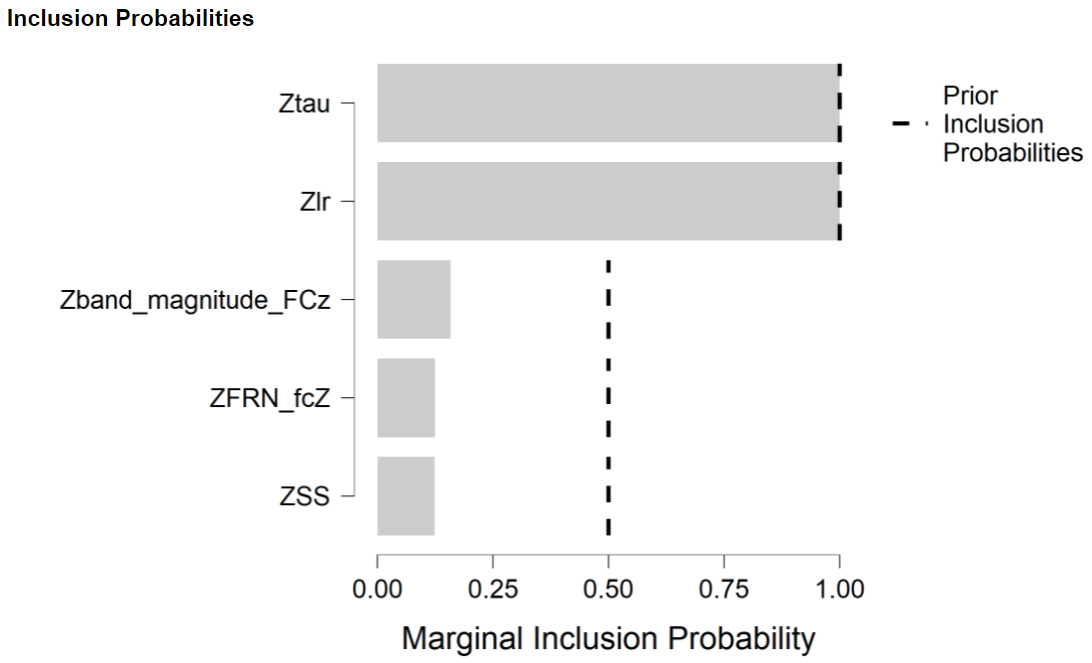


Study 2.2: the model excluded lr and tau and a null model as a reference


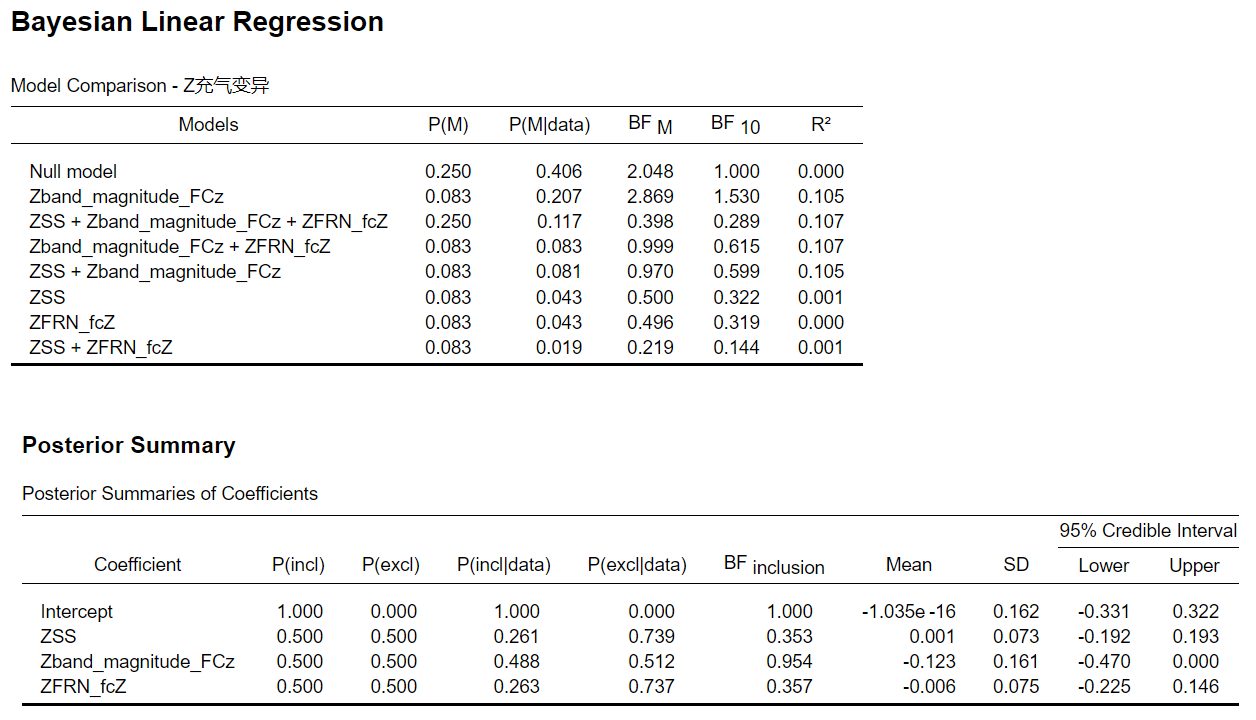


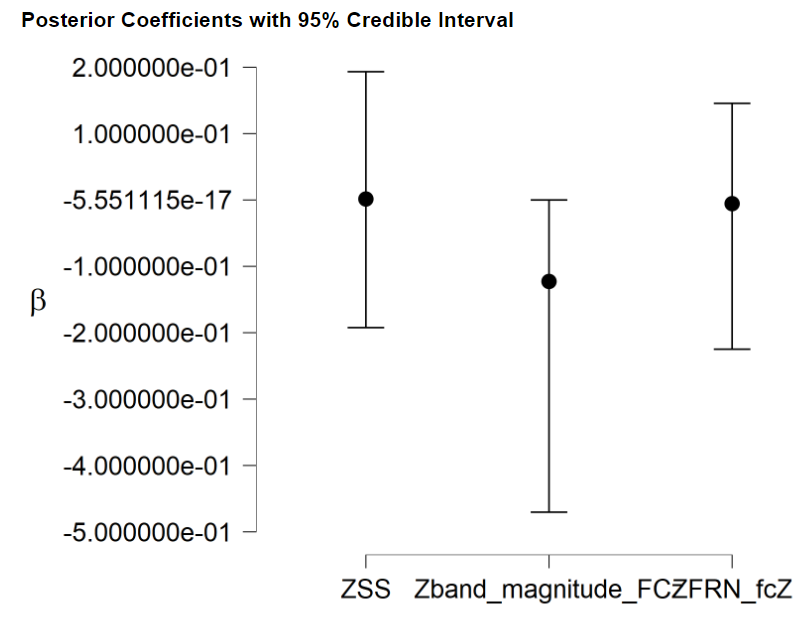

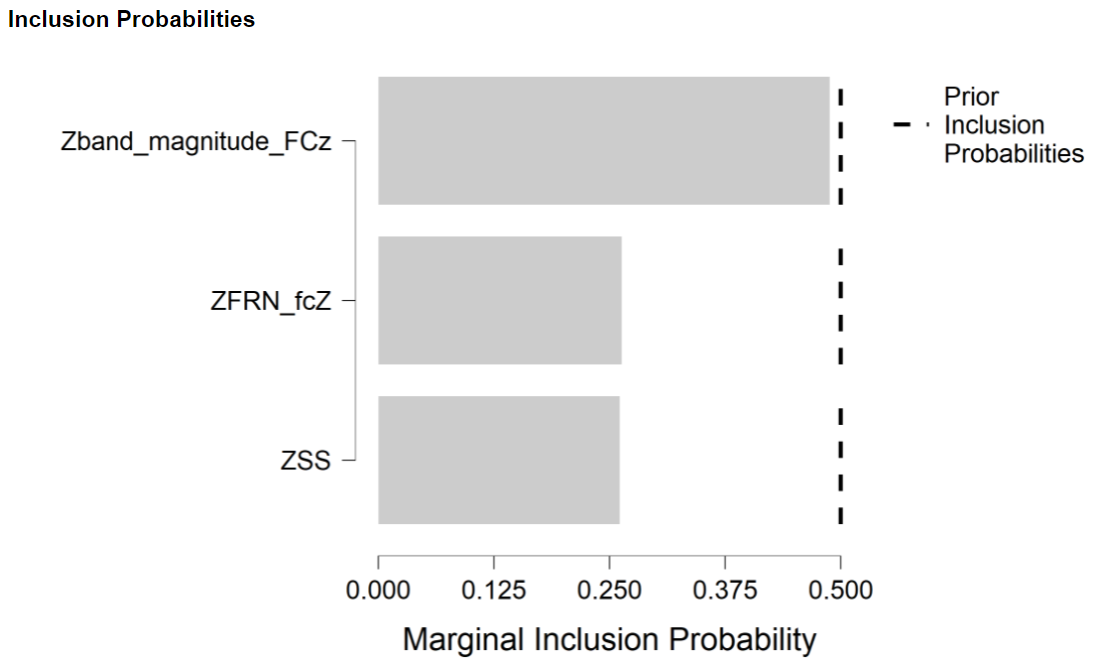

Supplement: Supplementary file 1 [file Table_1.DOCX]
